# Contrasting endemism in pond-dwelling cyclic parthenogens: the *Daphnia curvirostris* species group (Crustacea: Cladocera)

**DOI:** 10.1038/s41598-019-43281-9

**Published:** 2019-05-02

**Authors:** Alexey A. Kotov, Derek J. Taylor

**Affiliations:** 1 A. N. Severtsov Institute of Ecology and Evolution, Leninsky Prospect 33, Moscow, 119071 Russia; 2 Department of Biological Sciences, The State University of New York at Buffalo, Buffalo, NY 14260 USA

**Keywords:** Phylogenetics, Genetic markers

## Abstract

Pond-dwelling cyclic parthenogens are often proposed to be highly vagile. However, the Holarctic biogeography of parthenogens has been hampered by very limited sampling in the eastern Palearctic. Here we examine the geographic boundaries, diversity, and connectivity across the Palearctic for the *Daphnia curvirostris* complex (Cladocera: Daphniidae). Nuclear (HSP90) and mitochondrial (ND2) sequence data supported the existence of five main clades (most of which corresponded to presumptive species) with one eastern Palearctic clade being novel to this study (the average mitochondrial genetic divergence from known species was 19.2%). *D*. *curvirostris* s.s. was geographically widespread in the Palearctic, with a population genetic signature consistent with postglacial expansion. The Eastern Palearctic had local nine endemic species and/or subclades (other Holarctic regions lacked more than one endemic subclade). Even though several endemic species appeared to have survived Pleistocene glaciation in the eastern Palearctic, much of the Palearctic has been recolonized by *D*. *curvirostris* s.str. from a Western Palearctic refugium. A disjunct population in Mexico also shared its haplotypes with *D*. *curvirostris* s.str., consistent with a recent introduction. The only apparently endemic North American lineage was detected in a thermally disturbed pond system in northwestern Alaska. Our results for pond-dwelling cyclic parthenogens further support the hypothesis that the Eastern Palearctic is a diversity hotspot for freshwater invertebrates.

## Introduction

While the biogeography of terrestrial habitats has been studied for centuries^1^, our understanding of insular freshwater habitats remains poorly understood. Before genetic methods, freshwater animals were considered as exemplars of cosmopolitanism^2^. Later, the slogan *“Everything is everywhere*, *but the environment selects”* emerged^3^. For decades, cosmopolitanism reigned as the universal paradigm of freshwater biogeography^4^. Then, in the 1970’s, as cladocerans became developed as model organisms, non-cosmopolitanism gained favor^5,6^. But, with the recent discovery that freshwater anthropogenic introductions have been widespread^7–9^, the diagnosis of disjunct populations became problematic. Moreover, regional hotspots seemed to contain cryptic diversity for some taxa such as cladocerans^10^. The breeding system of most cladocerans, cyclic parthenogenesis, appeared to provide dispersal advantages. Ultimately, discerning recent introductions from relictual insular populations remains difficult without additional evidence from genetic markers. Fortunately, genetic analysis is now possible for testing biogeographic hypotheses for all sizes of freshwater animals.

The “ejected relict” scheme initially proposed by Wallace^1^ (see review by Eskov^11^), and later adapted for the freshwater Cladocera (Crustacea) by Korovchinsky^10^ has been used to explain latitudinal diversity centers. According to this hypothesis, a step-by-step mass extinction from the middle Caenozoic to recent times resulted from climate aridization. Refugia were located in the subtropics and the adjacent temperate zones in both the northern and southern hemispheres. Today these aridity refugia represent centers of cladoceran endemism: e.g. the Mediterranean zone, the Iberian Peninsula, and the southernmost extremity of Africa^10^.

Importantly, Pleistocene glacial oscillations have also had a strong regional effect on extinction and the current biogeography of Holarctic plants and animals^12^. Evidence for a biogeographic effect of glaciation in freshwater taxa has been reported in fishes^13–15^, freshwater insects^16^, molluscs^17^, and aquatic plants^18^. Similarly, cladoceran biogeography may have been markedly affected by Pleistocene glaciation in North America^19,20^ and in Europe^21,22^. However, large scale geographic studies are rare^23–25^ and some large regions such as the Eastern Palearctic are poorly studied^26–28^ compared to other regions of the Holarctic. Even less is known of the interactions of ecological and evolutionary processes that contributed to the biogeography of Eastern Palearctic cladocerans^29–32^.

Recently, evidence has emerged that the temperate Eastern Palearctic also represents a zone of cladoceran endemism^33^. Several endemics (or possible endemics) have been found here such as: *Macrothrix pennigera* Shen, Sung & Chen, 1961 in NE China^34^, *Ilyocryptus uenoi* Kotov & Tanaka, 2004 and *Diaphanosoma kizakiensis* Korovchinsky & Tanaka, 2013 in Japan^35,36^, *Daphnia sinevi* Kotov, Ishida & Taylor, 2006 and *Chydorus irinae* Smirnov & Sheveleva, 2010 in the Far East of Russia^37,38^ and *Pleuroxus jejuensis* Jeong, Kotov & Lee, 2013 in Korea^39^. Genetic evidence has been important in the establishment of the special zoogeographic status of the Eastern Palearctic^24,25,27,28^. Although some phyloclades are shared with the Western Palaearctic^30^, numerous endemic Eastern phyloclades have been detected. Some of these lineages are described as separate taxa^40,41^, but many others are waiting for such a description^27,42^. Kotov^33^ concluded that the Eastern Palearctic contains the highest degree of cladoceran endemism in the world.

Here we use genetic markers to test geographic boundaries of lineages in the widespread cladoceran, *Daphnia curvirostris* s.l. (Cladocera: Anomopoda: Daphniidae). Recently, three endemic species in the complex were described from the Palearctic: *Daphnia tanakai* Ishida, Kotov & Taylor, 2006 in Japan^40^, *Daphnia sinevi* Kotov, Ishida & Taylor, 2006 from Primorski Krai, Russia^37^, and *Daphnia hrbaceki* Juračka, Kořínek & Petrusek, 2010 from Central Europe^43^. We know little of the geographic boundaries of these species because there has been very limited geographic sampling.

Although the entire species complex is thought to be widespread in the temperate Palearctic (with rare occurrences in high altitude African lakes^44^), there are only four known sites from the Nearctic – all disjunct, see below. *D*. *curvirostris* was first reported in North America from three ponds in northwestern Canada^45^. It has not been reported from other locations in Canada since 1986. Duffy *et al*.^46^ then provided paleogenetic evidence that *Daphnia curvirostris* had invaded Onondaga Lake, New York and thrived from the 1950’s until the 1980’s when ephippia disappeared in the dated sediment record. The samples from NY showed 99% similarity in the mitochondrial 12SrRNA gene to sequences from two European specimens. A third disjunct population of *Daphnia curvirostris* was found in a thermally disturbed pond complex (Pilgrim Hotsprings) in northwest Alaska^40^. While clearly assignable to *Daphnia curvirostris*, the relationship of this Alaskan population to Palearctic populations remains unknown. Lastly, Nandini *et al*.^47^ recorded *Daphnia curvirostris* from Central Mexico and proposed an anthropogenic origin. However, there is presently no genetic comparison of the Mexican populations with potential sources in the Palearctic.

Here, we aim to better establish the geographic boundaries of the newly discovered Palearctic lineages and compare the disjunct Nearctic Mexican and Alaskan specimens to Palearctic specimens.

## Results

### Sequence variation

**Figure 1:**
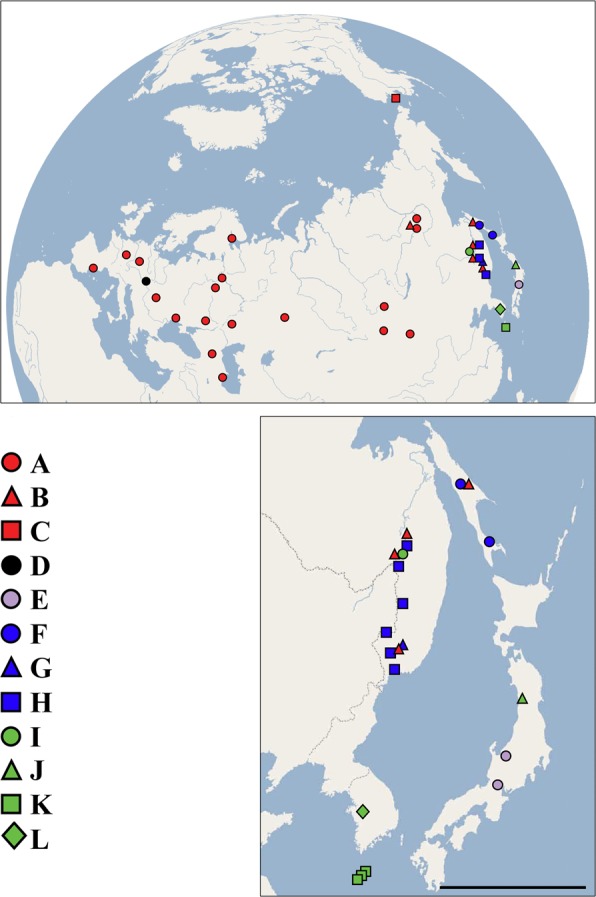
All sites from which diversity of the *Daphnia curvirostris* group has been analyzed (**a**), and a region of the group maximum diversity — Far East (**b**). The base map is the Marble Virtual Globe 1.5.1 “plain map” (i.e., no attributable data layers) available at https://marble.kde.org/.

We collected genetic information for 194 individuals of the *D*. *curvirostris* complex from 51 populations across the Palearctic with a special focus on the Eastern Palearctic (Fig. 1; Supplementary Table S1).

**Figure 2:**
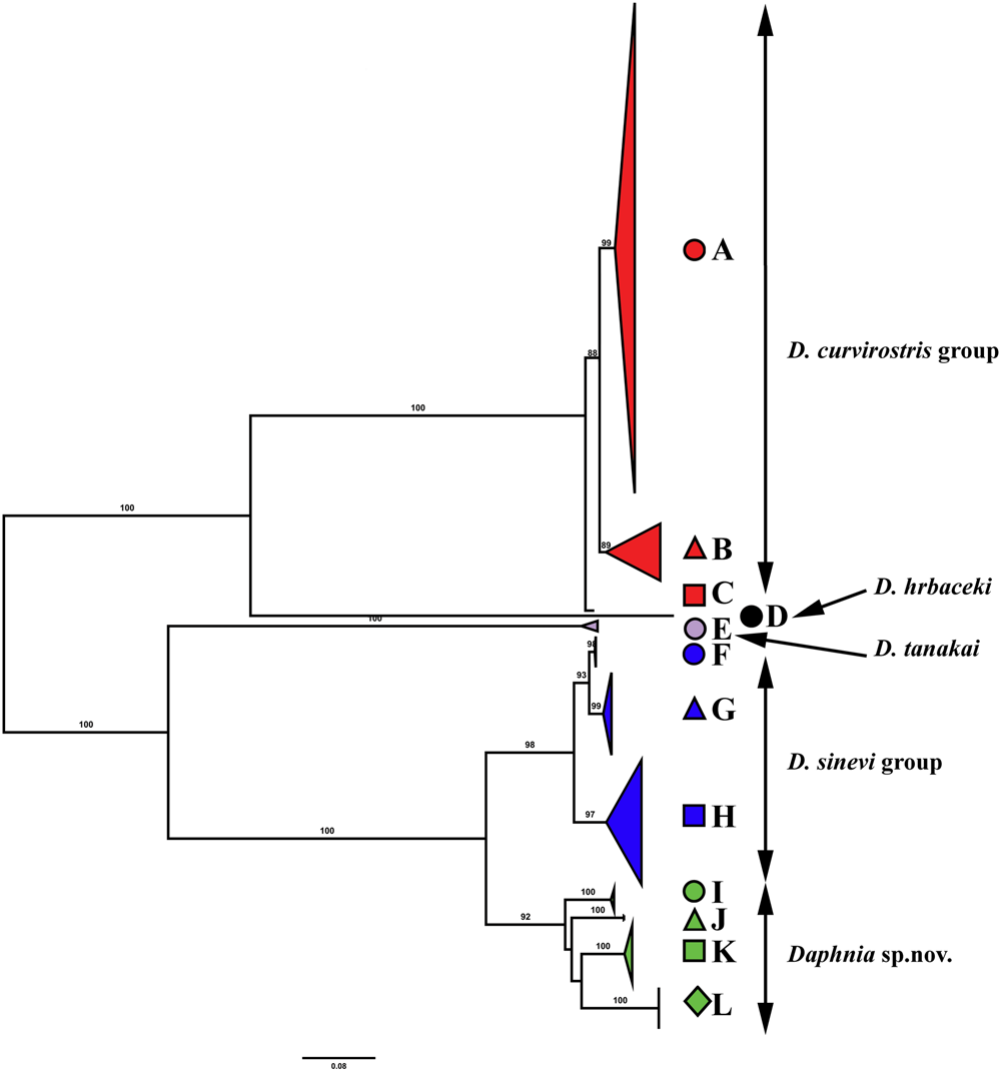
Maximum likelihood tree based on sequences of the mitochondrial ND2 gene representing the diversity among phylogroups of the *Daphnia curvirostris* group. The support values of individual nodes are based on maximum likelihood. Colors and shapes for main phylogroups correspond to those in Fig. 1.

**Figure 3:**
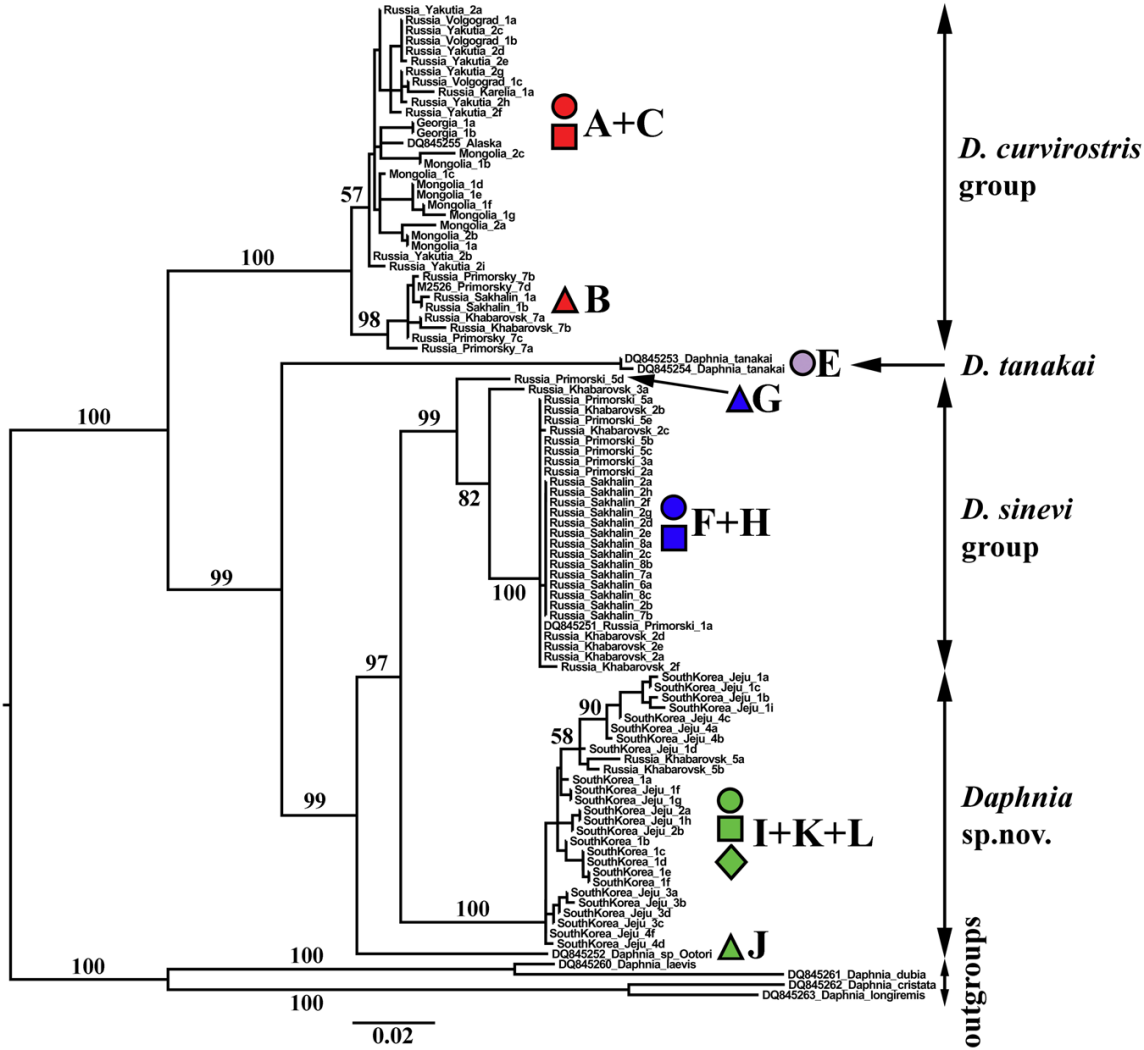
Maximum likehood tree based on sequences of nuclear HSP90 gene representing the diversity among clades of *Daphnia curvirostris* group. The support values of individual nodes are based on maximum likelihood. Colors and shapes correspond to mitochondrial phylogroups in Figs 1 and 2.

The alignment of *ND2* (mitochondrial NADH dehydrogenase 2) was unambiguous and each sequence had a single open reading frame. Only single codon deletions were observed within the complex. This deletion was present in a 10–codon long variable region of an Alaskan sequence that also had differing single codon deletions in the outgroup species and in *D*. *hrbaceki*. The best fit model for the ingroup *ND2* alignment was TN + F + I + G4 which had a Bayesian Information Criterion score of 23451.8840. All but one of our analyses found the same root for the *D*. *curvirostris* complex (Figs 2 and 3; Supplementary Figs S1–S3). For the *ND2* alignments, three outgroup sequences for the nucleotide data failed the base composition homogeneity test provided by IQtree while no outgroup sequences for the amino acid data failed the same test.

### *ND2* phylogenetic tree and geographic distributions

The entire *D*. *curvirostris* complex was monophyletic and divided into five major clades which may represent species or species groups (Fig. 2). Major clade 1 (*Daphnia curvirostris* s.str.) had three geographic subclades (subclades are lettered from A-C, red in Figs 1 and 2). Subclade A had a broad distribution in the Northern Palearctic from France to Eastern Siberia (Yakutia; Fig. 1). The Mexican specimens were nested within subclade A, supporting the Palearctic origin hypothesis. A more basal Subclade B within *D*. *curvirostris* was detected in Eastern Siberia (Yakutia), the continental portion of the Far East of Russia and Sakhalin Island (but absent from the Japanese samples). Another basal lineage was subclade C which was found only in a single locality (Pilgrim Hotsprings) in northwestern Alaska. Major clade 2 (*D*. *hrbaceki*) contained the type locality population (a recently created pool in Central Europe) of *D*. *hrbaceki* (subclade D, black circle in Fig. 2). The remaining major clades were restricted to the eastern Palearctic. Major clade 3 (also a described species, *D*. *tanakai*) was represented by a single subclade E (grey circle in Fig. 2), and was restricted to mountain lakes in Japan. Major clade 4 (*D*. *sinevi*, blue in Fig. 2) was ranged from the continental Far East of Russia to Sakhalin Island. Clade 4 contained three more regionally distributed subclades: subclade F was endemic to Sakhalin Island; subclade G was found in a single population in Primorie (the continental Far East of Russia); and subclade H was relatively widely distributed in the continental Far East of Russia. Major clade 5 (termed *Daphnia* sp. nov. here to denote a putative new species, green in Fig. 2) was present in the continental Far East of Russia, Japan and Korea, and was represented by four subclades, each with very restricted ranges: subclade I was found in a single locality on the continental Far East of Russia, the subclade J was found in a single lake in Japan, subclade K was present in a single locality in continental Korea, and subclade L was found in four localities on Jeju Island (South Korea). Major Clade 5 differed from the closest clade with an existing species name (*D*. *sinevi*) by an average of 19.2% for the ND2 sequence using a Jukes-Cantor corrected distance (Table 1).

**Table 1:** Average number of nucleotide substitutions per site with Jukes Cantor correction (Dxy(JC)) between taxa of the *Daphnia curvirostris* complex.

Taxon or clade	*curvirostris*	*hrbaceki*	*sinevi*	*tanakai*	*Daphnia* sp. nov. complex
**Curvirostris subclade A (96)**					
*hrbaceki*	0.388 (0.095)				
*sinevi* (49)	0.512 (0.031)	0.476 (0.179)			
*tanakai* (3)	0.476 (0.071)	0.464 (0.219)	0.380 (0.086)		
*Daphnia* sp. nov. (28)	0.494 (0.034)	0.426 (0.209)	0.192 (0.01987)	0.346 (0.102)	
*curvirostris* AK	0.035 (0.009)	0.390	0.468 (0.176)	0.478 (0.225)	0.460 (0.225)

Numbers in parentheses are the standard deviations; numbers in parentheses beside taxon names are sample size. *D*. *curvirostris* from Alaska (AK) is shown separately from Palearctic sequences.

The protein based ND2 trees (Supplementary Figs S2 and S3) were very similar to the nucleotide tree with strong support for the five major clades and most of the minor subclades. One difference between the protein and nucleotide based trees was the switched positioning of the Alaskan *D*. *curvirostris* with the subclade B sequences – but both B and C remained basal to the geographically widespread A subclade of *D*. *curvirostris*. Notably, outgroup rooting with an amino acid alignment (Supplementary Figs S2 and S3) supported the same root for the *curvirostris* complex as found by the midpoint rooted nucleotide tree (Fig. 2).

### HSP90 tree

Model-fitting determined three models for the partitions by codon position and intron/exon. For the first and second positions, TN + F + I was the best fit model, while K2P + G4 was the best fit model for third codon positions. For introns the best fit model was HKY + F + G4. The well supported branches of the HSP90 and ND2 trees were in agreement (Fig. 3). Five major clades with strong support were found (note that we lacked HSP90 sequences for *D*. *hrbaceki*, the major clade 2 of mitochondrial tree). Again, major clade 1 corresponded to the *Daphnia curvirostris* group. It contained two subclades, corresponding to the mitochondrial clades A + C (with moderate support) and clade B (with a strong support). Major clade 2 corresponded to *D*. *tanakai*, represented by sequences from two lakes in Japan. It contained no structure and corresponded to mitochondrial subclade E. Major clade 3 corresponded to *D*. *sinevi*. It contained two clades, corresponding to mitochondrial subclades G, and F + H. Major clade 4 corresponded to a single mitochondrial subclade J, a portion of the undescribed species complex *Daphnia* sp. nov. Major clade 5 corresponded to the rest of this proposed new species complex, mitochondrial subclades I + K + L. As with ND2, *D*. *curvirostris*-like taxa form a well-supported monophyletic clade with the same root.

### ND2 median joining (MJ) haplotype network for *Daphnia curvirostris* s.str

**Figure 4:**
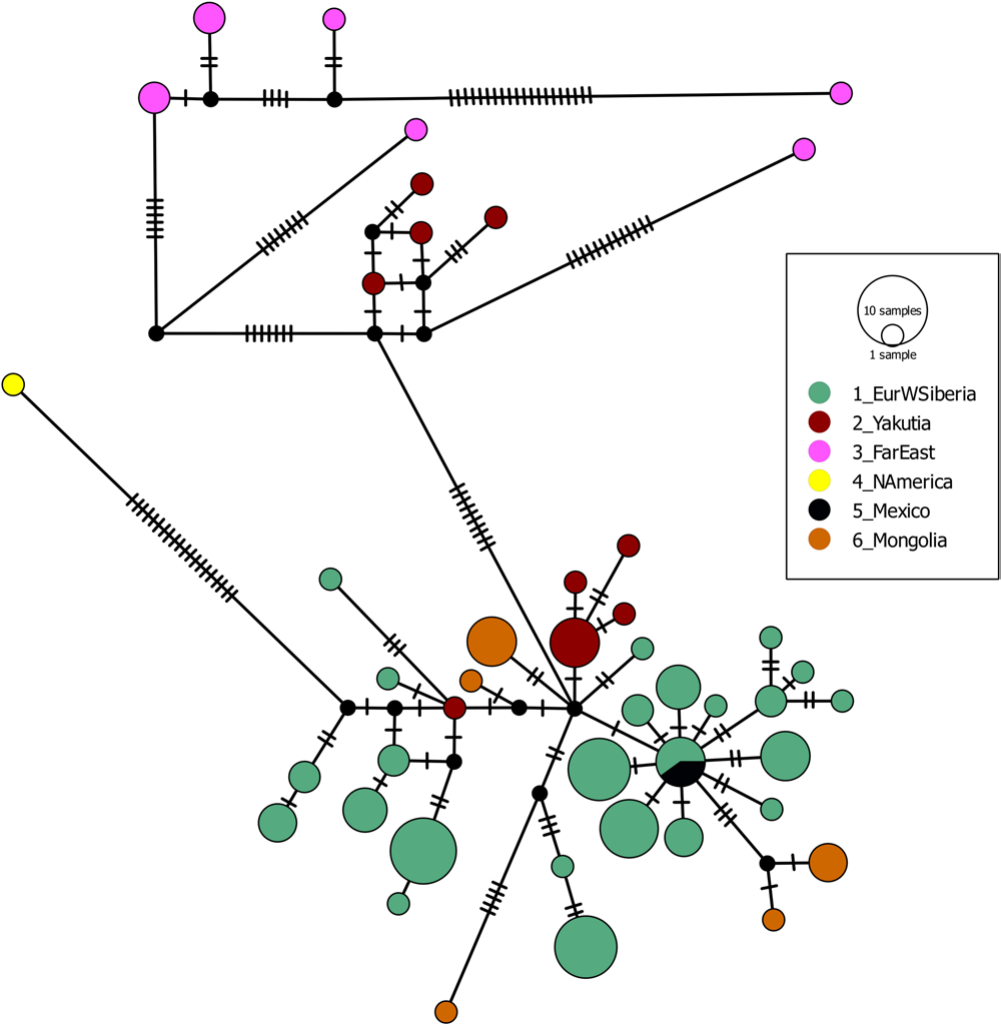
Median-joining *ND2* haplotype network. Median vectors are indicated by small black circles. Number of mutations for each branch is represented.

The MJ network of *Daphnia curvirostris* contained 40 different ND2 haplotypes (Fig. 4). The three main divisions (A-C) found in the tree analyses for *D*. *curvirostris* were apparent in the network.

Subclade A was represented by a sub-network of closely-related haplotypes (the average number of nucleotide differences k = 3.8). Still only private haplotypes (no regional sharing) for the eastern and western Palearctic were observed. There was at least one star-like topology, suggesting recent population expansion. The central haplotype of this star contained European-Western Siberian and Mexican individuals (Tajima’s D = −1.55; P = 0.048; Fu’s Fs = −14.57; P < 0.001). Subclade B was represented by a group of haplotypes that lacked a star-like pattern (Tajima’s D = −0.65072, P > 0.10; Fu’s Fs = −1.08), with modest haplotype divergence (average number of nucleotide differences, k = 19.6). Subclade C from Alaska was represented by a single haplotype connected to a haplotype within the clade A, but differed from subclade A by an average of 19.1 nucleotides.

The mismatch distribution analyses (Supplementary Fig. S4) for subclade A deviated significantly from the graph expected by constant population size (raggedness index = 0.0126; P = 0.017).

In order to assess the potential for mitochondrial pseudogenes in our data we translated the codon sequences and tested for pervasive purifying selection (an expected pattern for mitochondrial genes). The prevailing pattern of selection for ND2 in the *D*. *curvirostris* complex was purifying selection (non-synonymous/synonymous rate ratio = 0.1299). However, MEME did identify eight sites with significant episodic positive selection (Supplementary Fig. S5). A similar pattern was found with FUBAR (Supplementary Table S2) where four sites had significant episodic positive selection and 206 sites had significant purifying selection.

## Discussion

The results indicate that the present genetic diversity of the geographically widespread *Daphnia curvirostris* complex is greatest in the eastern Palearctic. However, we did find evidence for a widely distributed clade that appears to have expanded from a single glacial refugium to a large portion of the Palearctic (with recent, presumably anthropogenic introduction to Mexico).

Often, the present ranges of Holarctic freshwater animals are proposed to result from extinction and bottlenecks during the last glacial maximum followed by expansion from multiple glacial refugia^12^. The present-day contact zones of these lineages is often detected at a considerable distance from the refugia. In the Palearctic, for example, a contact zone has been detected in central Siberia for several cladocerans^30–32^. Here, the genetic results suggest that the *Daphnia curvirostris* complex contains a single geographically widespread (Palearctic and beyond) subclade A and several divergent Eastern Palearctic clades and species (including putative novel species). The geographic limit of *D*. *curvirostris* s.str. (Clade 1) appears to be near the eastern extremity of the Palearctic in the Lena River basin (Yakutia) – no shared haplotypes were detected across this boundary.

Also unusual for cladocerans (but see^48^) is the observation that only one clade (that appears to emanate from a single refugium) for the *D*. *curvirostris* complex has a broad geographic distribution. The star-like network and significantly negative Tajima’s D and Fu’s Fs for this clade are consistent with a recent population expansion. The raggedness r statistic indicates that the mismatch distribution has a significantly different shape from that expected from constant size (but is consistent with population growth). The population structure does not appear to be limited to mitochondrial DNA as we detected very similar patterns (albeit with less divergence) in the nuclear encoded introns of HSP90 (Fig. 3). Nor does our assessment of variation in the population variation of mtDNA in the eastern Palearctic populations appear to be contaminated with pseudogenes. We detected no stop codons and the codons are under strong purifying selection as would be expected from a functional mitochondrial protein-coding gene. Although we lack a reliable method of calibrating the molecular clock for *Daphnia curvirostris*, we note that the genetic divergences and star-like patterns for ND2 (subclade A) are very close to those found for the multiple proposed postglacial expansions in *Daphnia galeata*, *Daphnia dentifera*, and *Bosmina longispina* using the same gene^24,25,49^. Because the distribution is in a region that was profoundly affected (or covered) by glaciation during the LGM, and normally only the most recent glacial expansions can be detected with contemporary data, the data are consistent with an expansion time that is post-LGM^50,51^.

We observed one difference in rooting involving the position of *D*. *tanakai*, which moved to a more basal in the nucleotide-based tree (Supplementary Fig. S1). However, the evidence suggests that a basal position of *D*. *tanakai* may result from a systematic bias (such as long branch attraction to the outgroups). First, three of the outgroups failed a base composition heterogeneity test which would complicate ML analyses with a single substitution model. Removal of the outgroups (using midpoint rooting) placed the root in the same position as the nuclear gene tree. Notably, the more slowly evolving amino acid tree for ND2 (with outgroup rooting) agreed with the nuclear gene tree in root position (even after adding more closely related outgroup species). Lastly, the phylogenetic grouping of *D*. *tanakai* with the other far eastern Palearctic lineages is a biogeographically simpler explanation than a basal position of *D*. *tanakai*.

Unlike in other *Daphnia*^52^, the widespread subclade of *D*. *curvirostris* does not appear to originate in the eastern Palearctic. Japan has been regarded as a diversity source for several widespread cladocerans lineages^24,25,52^. However, some mitochondrial lineages appear to have colonized Japan from more northerly regions^31,32^. Why some lineages of *Daphnia* have recently expanded over vast distances while others remain restricted to a single or a few sites remains a mystery.

The finding that the Mexican population shares the identical central mitochondrial haplotype for the widespread Palearctic subclade of *D*. *curvirostris* indicates recent connectivity with Palearctic populations. The finding is consistent with the recent introduction of the disjunct Mexican *D*. *curvirostris* from Europe. As there are no natural dispersal vectors for *Daphnia* between the Palearctic and Mexico, the simplest explanation is anthropogenic introduction^53,54^. Duffy *et al*.^46^ made a similar proposal for the now extirpated population of *D*. *curvirostris* in Western New York. Unlike the other North American *D*. *curvirostris*, the Alaskan *D*. *curvirostris* had a unique codon deletion and was differentiated from the widely distributed clade of *D*. *curvirostris*. So, there is some evidence that this Pilgrim Hotsprings population may be unique and endemic. Other studies have failed to detect *D*. *curvirostris* in Alaska beyond the thermally-disturbed region associated with Pilgrim Hot Springs despite extensive sampling in western Alaska. More sampling will be needed to determine if other thermally and conductivity disturbed regions in the western Nearctic contain rare genetically differentiated populations in the *D*. *curvirostris* group. It is plausible that hot springs in Beringia have acted as microrefugia for temperate freshwater species during the LGM.

The greater diversity of the *Daphnia curvirostris* species group in the temperate Eastern Palearctic compared to the Western Palearctic is a common geographic pattern in plants and animals^55^. Even before the modern era of genetics, endemic cladocerans^35,36^ and other invertebrates^56,57^ were known from the eastern Palearctic. Reduced extinction during the Pleistocene glacial cycles is normally proposed as a contributing factor. The relatively moderate climate of the Eastern Palearctic compared to regions directly affected by Pleistocene ice sheets may have been the most important factor affecting extinction rates^58^. Even so, the Eastern and Western Palearctic were far from homogenous regions during the LGM. During the LGM, nunataks and even patches of forest existed in the Western Palearctic while regions of harshness and smaller glaciers (montane regions) existed in the Eastern Palearctic.

*Daphnia curvirostris* has been proposed as a temperate species complex with a distribution limited by temperature requirements^8^. In the eastern Palearctic, *D*. *curvirostris*-like specimens have been detected in North China (the exact location is unknown, see^34,59^. However, in the true Asian tropics^60,61^ and subtropics (i.e. in Hainan Island^62^) *D*. *curvirostris* remains undetected. It is unlikely that any of the lineages that we detected are common in the tropics. Most of the lineages belong to a relatively narrow region close to the Japan and Yellow Sea (including Sakhalin Island, the Korean Peninsula, Japan and NE China (about 30–50°N, 120–145°E). We conclude that this temperate geographic region is a hotspot of the biodiversity and a zone of endemism for *curvirostris*-like taxa (and perhaps other cladocerans). The explanation for this geographic diversity remains to be addressed – but both extinction and speciation appear to have played a role^10^. Speciation may explain the pronounced endemism and regionalism within the “hot spot”.

Speciation seems to have played a role because the *D*. *curvirostris* complex has very strong sub-regional differentiation: there is no subclade sharing among Japan, the Russian Far East, continental Korea or Jeju Island. A single subclade only is shared between the continent and Sakhalin Island, while another Sakhalinian clade is an endemic of the island. One interesting location for endemics is the mountains of the largest Japanese island, Honshu. Subclade J (*Daphnia* sp. nov.) was undetected in other water bodies of Japan (or any other region), although about 100 lakes and ponds were sampled and populations of different species of *Daphnia* were found in many water bodies of Japan^24,25,42,51^. So, this subclade (and likely a new biological species, see the HSP90 tree) may be present in very few water bodies on the planet. One possible geographic speciation scenario for the origin of species in the eastern Palearctic is the disruption of a continuous land mass, connecting Korea Peninsula, Japan, Sakhalin and continental Far East of Russia^63,64^. The same biogeographic pattern is known for several other freshwater and terrestrial animals^57,65^.

Our study indicates that there is more diversity in the *Daphnia curvirostris* complex than previously appreciated. We find an amalgam of several lineages of differing divergences in this complex, with one lineage having a broad Palearctic distribution and 10 others being endemic to parts of the Eastern Palearctic. Thus, the effect of the LGM and Holocene deglaciation has been very different within a freshwater species complex. This study represents the first step towards understanding the global biogeography and diversity of the *Daphnia curvirostris* complex. The new species detected here will be formally described in a taxonomic paper. Although local extirpation of populations of the geographically widespread clade (as happened in New York) will represent a negligible loss in diversity, losses of single populations in the Eastern Palearctic (or from thermally disturbed pools) could represent the loss of a divergent lineage or even a species.

Empirical studies have often shown that relicts are particularly susceptible to extinction^66,67^. Moreover, there are ongoing threats to small freshwater habitats such as eutrophication, drainage, and anthropogenic introductions^9,68,69^. As such, we consider the study of cladoceran biology and conservation in the eastern Palearctic and subarctic hotsprings to be priorities.

## Methods

### Ethics statement

No species in this study are listed as protected or endangered. Field collection in Russia was carried out by our team or by colleagues as part of a governmental project “Ecology and biodiversity of aquatic ecosystems and invasions of alien species” (No. 0109-2014-0008), with governmental permission to collect samples from public property. Sampling in the natural reserves of Russia was conducted with special permissions of their Directors. Verbal permission to collect in private farm ponds was obtained from local owners. Mongolian samples were collected by the Joint Russian-Mongolian Complex Biological Expedition with permission of the Ministry of Nature, Environment and Tourism of Mongolia. Field collections in South Korea were carried out in frames of fulfillment of the program from the National Institute of Biological Resources of Korea. Some samples from other countries were provided by colleagues who collected them with permissions linked to their activity as hydrobiologists in governmental institutions of their countries, where the collection of these invertebrates is not subjected to restriction by national or international laws and does not require special permission.

### Field collection and DNA extraction

Specimens were collected by plankton nets with diameter of 20–40 cm and mesh size of 30–50 µm, or rectangular dip nets of same mesh size with width of 0.2–0.3 m, handle length of 0.5–2 m. After collection specimens were in 96% alcohol. Before the start of the genetic studies, each specimen was preliminarily identified by morphological characters^37,40,44^. The majority of samples are Palearctic but we also included specimens from disjunct Nearctic populations (Mexico and Alaska). DNA was extracted from single adult females using the DNA Quickextract (Epicentre) protocol as modified by Ishida *et al*.^40^.

### Sequencing and alignment

PCR was carried out using the Promega GoTaq master reaction mix (PCR cycling conditions were 95 °C for 2 m, 95 °C for 30 s, 48 °C for 30 s, and 72 °C for 1 m for 39 cycles, followed by 72 °C for 5 m). We used *Daphnia*-specific primers^40^ for the mitochondrial ND2 gene (approximately 960 bp) and the nuclear HSP90 gene (approximately 700 bp). After gel verification of amplicon size, PCR products were purified and Sanger sequenced (TACGEN, California). Sequence chromatograms were assembled and examined in Geneious R7^70^. Each amplicon was sequenced in both directions and the assembled reads were examined manually for internal peaks and translation. Primer regions were trimmed and consensus sequences were generated based on highest quality. The nuclear gene amplicon contained two introns. Alignment was carried out using Muscle as implemented in Seaview 4.6^71^. Translation alignment (to preserve the codon structure) was carried out by aligning amino acids and then reverting to nucleotides in Seaview. Intron boundaries were determined according to Ishida *et al*.^40^.

### Phylogenetics

Substitution model fitting was carried out in IQtree 1.6^72^ with the number of partitions optimized by codon position and intron/exon. Tree estimation used the maximum likelihood algorithm of IQtree and the best fit partition model^73^. Branch support was estimated with nonparametric bootstrapping and approximate likelihood ratio tests (aLRT) with 1000 replicates. Sequences of four species (*Daphnia laevis*, *Daphnia dubia*, *Daphnia longiremis*, and *Daphnia cristata*) were used as outgroups. The effect of adding more closely related (to the ingroup *D*. *curvirostris* complex) outgroups (*D*. *galeata* and *D*. *umbra*) was also explored. Phylograms were visualized in Figtree 1.43^74^. Protein based phylogenies were estimated using PhyML 3.0 as implemented by Seaview 4.6 using the arthropod-mitochondrial (mtart) + gamma substitution model.

To explore geographic structure in populations of *Daphnia curvirostris* sensu stricto, we estimated median joining networks in PopArt v1.7 beta^75^. Tests of selection for the ND2 gene were carried out using Fast Unconstrained Bayesian Approximation (FUBAR^76^) and Mixed Effects Model of Evolution (MEME^77^) as implemented in Datamonkey 2.0. Mismatch analyses, coalescent simulations and statistics associated with population growth and divergence were carried out using DnaSP 6^78^.

## Supplementary information

Supplementary information to the paper: Contrasting endemism in pond-dwelling cyclic parthenogens: the Daphnia curvirostris species group (Crustacea: Cladocera)

## Data Availability

Voucher specimens are deposited into AAK private collection. DNA sequences were submitted to Genbank resulting in the following accession numbers (MH613987 – MH614169 and MH733953 – MH734039).

## References

[1] Wallace, A. R. The geographical distribution of animals (Harper; 1876).

[2] Darwin, C. The origin of species by means of natural selection, or, the preservation of favoured races in the struggle for life. (Grant Richards; 1902). PMC5184128 30164232

[3] Baas Becking, L. G. M. Geobiologie of inleiding tot de Milieukunde. 1–263 (Van Stockum W. P. & Zoon, 1934).

[4] Brehm V (1955). Süsswasserfauna und Tiergeographie. Őst. zool. Zeitsch..

[5] Frey DG (1982). Questions concerning cosmopolitanism in Cladocera. Arch. Hydrobiol..

[6] Frey DG (1987). The taxonomy and biogeography of the Cladocera. Hydrobiologia.

[7] Briski E, Cristescu M, Bailey S, MacIsaac H (2010). Use of DNA barcoding to detect invertebrate invasive species from diapausing eggs. Biol. Invasions.

[8] Mergeay J, Verschuren D, De Meester L (2005). Cryptic invasion and dispersal of an American *Daphnia* in East Africa. Limnol. Oceanogr..

[9] Karabanov DP, Bekker EI, Shiel RJ, Kotov AA (2018). Invasion of a Holarctic planktonic cladoceran *Daphnia galeata* Sars (Crustacea: Cladocera) in the Lower Lakes of South Australia. Zootaxa.

[10] Korovchinsky NM (2006). The Cladocera (Crustacea: Branchiopoda) as a relict group. Zool. J. Linn. Soc..

[11] Eskov, K. Y. Continental drift and problems of historical biogeography in *Faunogenez i filocenogenez* (ed. Chernov, Y. I.) 24–92 (Nauka, 1984).

[12] Hewitt GM (2000). The genetic legacy of the Quaternary ice ages. Nature.

[13] Bernatchez L, Wilson CC (1998). Comparative phylogeography of Nearctic and Palearctic fishes. Mol. Ecol..

[14] Makhrov AA, Bolotov IN (2006). Dispersal routes and species identification of freshwater animals in Northern Europe: a review of molecular evidence. Russ. J. Genet..

[15] April J, Hanner RH, Dion-Côté AM, Bernatchez L (2013). Glacial cycles as an allopatric speciation pump in north-eastern American freshwater fishes. Mol. Ecol..

[16] Theissinger K (2013). Glacial survival and post‐glacial recolonization of an arctic–alpine freshwater insect (*Arcynopteryx dichroa*, Plecoptera, Perlodidae) in Europe. J. Biogeogr..

[17] Bolotov IN (2017). Origin of a divergent mtDNA lineage of a freshwater snail species, Radix balthica, in Iceland: cryptic glacial refugia or a postglacial founder event?. Hydrobiologia.

[18] Eidesen PB (2013). Genetic roadmap of the Arctic: plant dispersal highways, traffic barriers and capitals of diversity. New Phytol..

[19] Taylor DJ, Finston TL, Hebert PDN (1998). Biogeography of a widespread freshwater crustacean: Pseudocongruence and cryptic endemism in the North American *Daphnia laevis* complex. Evolution.

[20] Cox, A. J. Freshwater phylogeography: the impact of life history traits on the post-glacial dispersal of zooplankton in North America. Mr. Sci. Thesis. (The University of Guelph; 2001).

[21] De Gelas K, De Meester L (2005). Phylogeography of *Daphnia magna* in Europe. Mol. Ecol..

[22] Hamrová E, Krajicek M, Karanovic T, Černý M, Petrusek A (2012). Congruent patterns of lineage diversity in two species complexes of planktonic crustaceans, *Daphnia longispina* (Cladocera) and *Eucyclops serrulatus* (Copepoda), In East European mountain lakes. Zool. J. Linn. Soc..

[23] Crease TJ, Omilian AR, Costanzo KS, Taylor DJ (2012). Transcontinental phylogeography of the *Daphnia pulex* species complex. PLoS One.

[24] Ishida S, Taylor DJ (2007). Quaternary diversification in a sexual Holarctic zooplankter, *Daphnia galeata*. Mol. Ecol..

[25] Ishida S, Taylor DJ (2007). Mature habitats associated with genetic divergence despite strong dispersal ability in an arthropod. BMC Evol. Biol..

[26] Belyaeva M, Taylor DJ (2009). Cryptic species within the *Chydorus sphaericus* species complex (Crustacea: Cladocera) revealed by molecular markers and sexual stage morphology. Mol. Phyl. Evol..

[27] Xu S, Hebert PDN, Kotov AA, Cristescu ME (2009). The non-cosmopolitanism paradigm of freshwater zooplankton: insights from the global phylogeography of the predatory cladoceran *Polyphemus pediculus* (Crustacea, Onychopoda). Mol. Ecol..

[28] Xu L (2010). Biogeography and evolution of the Holarctic zooplankton genus *Leptodora* (Crustacea: Branchiopoda: Haplopoda). J. Biogeogr..

[29] Ma X (2015). Diversity of the *Daphnia longispina* species complex in Chinese lakes: a DNA taxonomy approach. J. Plankt. Res..

[30] Bekker EI, Karabanov DP, Galimov YR, Kotov AA (2016). DNA barcoding reveals high cryptic diversity in the North Eurasian *Moina* Species (Crustacea: Cladocera). PLoS One.

[31] Bekker EI (2018). Phylogeography of *Daphnia magna* Straus (Crustacea: Cladocera) in Northern Eurasia: Evidence for a deep longitudinal split between mitochondrial lineages. Plos One.

[32] Kotov AA, Karabanov DP, Bekker EI, Neterina TV, Taylor DJ (2016). Phylogeography of the *Chydorus sphaericus* group (Cladocera: Chydoridae) in the Northern Palearctic. PLoS One.

[33] Kotov AA (2016). Faunistic complexes of the Cladocera (Crustacea, Branchiopoda) of Eastern Siberia and Far East of Russia. Zool. Zh..

[34] Chiang, S. C. & Du, N. S. Freshwater Cladocera. Fauna Sinica. Crustacea. 1–297. (Science Press, Academia sinica, 1979).

[35] Kotov AA, Tanaka S (2004). *Ilyocryptus uenoi* sp. nov. (Anomopoda, Cladocera, Branchiopoda) from Japan. Hydrobiologia.

[36] Korovchinsky NM (2012). A new species of the genus *Diaphanosoma* Fischer, 1850 (Crustacea: Cladocera: Sididae) from Japan. Limnology.

[37] Kotov AA, Ishida S, Taylor DJ (2006). A new species in the *Daphnia curvirostris* (Crustacea: Cladocera) complex from the eastern Palearctic with molecular phylogenetic evidence for the independent origin of neckteeth. J. Plankt. Res..

[38] Smirnov NN, Sheveleva NG (2010). *Chydorus irinae* sp. n. (Anomopoda, Chydoridae, Chydorinae) from the Tom’ River (the Amur basin, Russia). Zool. Zh..

[39] Jeong HG, Kotov AA, Lee W (2014). Checklist of the freshwater Cladocera (Crustacea: Branchiopoda) of South Korea. Proc. Biol. Soc. Wash..

[40] Ishida S, Kotov AA, Taylor DJ (2006). A new divergent lineage of *Daphnia* (Cladocera: Anomopoda) and its morphological and genetical differentiation from *Daphnia curvirostris* Eylmann, 1887. Zool. J. Linn. Soc..

[41] Kotov AA, Ishida S, Taylor DJ (2009). Revision of the genus *Bosmina* Baird, 1845 (Cladocera: Bosminidae), based on evidence from male morphological characters and molecular phylogenies. Zool. J. Linn. Soc..

[42] Ishida S (2011). The long-term consequences of hybridization between the two *Daphnia* species, *D*. *galeata* and *D*. *dentifera*, in mature habitats. BMC Evol. Biol..

[43] Juračka PJ, Kořínek V, Petrusek A (2010). A new Central European species of the *Daphnia curvirostris* complex, *Daphnia hrbaceki* sp. nov. (Cladocera, Anomopoda, Daphniidae). Zootaxa.

[44] Mergeay J, Verschuren D, De Meester L (2005). *Daphnia* species diversity in Kenya, and a key to the identification of their ephippia. Hydrobiologia.

[45] Hebert PDN, Loaring JM (1986). Systematics of the *Daphnia pulex* group: variation in an agamic complex and description of a species new to North America. Biochem. Syst. Ecol..

[46] Duffy MA, Perry LJ, Kearns KM, Weider LJ, Hairston NG (2000). Paleogenetic evidence for a past invasion of Onondaga Lake, New York, by exotic *Daphnia curvirostris* Using mtDNA from Dormant Eggs. Limnol. Oceanogr..

[47] Nandini S (2009). First record of the temperate species *Daphnia curvirostris* Eylmann, 1887 emend. Johnson, 1952 (Cladocera: Daphniidae) in Mexico and its demographic characteristics in relation to algal food density. Limnology.

[48] Popova EV (2016). Revision of the Old World *Daphnia* (*Ctenodaphnia*) *similis* group (Cladocera: Daphniidae). Zootaxa.

[49] Haney RA, Taylor DJ (2003). Testing paleolimnological predictions with molecular data: the origins of Holarctic *Eubosmina*. J. Evol. Biol..

[50] Grant WS (2015). Problems and cautions with sequence mismatch analysis and Bayesian skyline plots to infer historical demography. J. Hered..

[51] Weydmann A (2018). Postglacial expansion of the Arctic keystone copepod *Calanus glacialis*. Mar. Biodiver..

[52] Ishida, S. The effects of Quaternary glacial cycles on the evolution of Holarctic *Daphnia*. Ph. D. Thesis. (State University of New York at Buffalo; 2007).

[53] Havel JE, Hebert PDN (1993). *Daphnia lumholtzi* in North America: Another exotic zooplankter. Limnol. Oceanogr..

[54] Havel JE, Colbourne JK, Hebert PDN (2000). Reconstructing the history of intercontinental dispersal in *Daphnia lumholtzi* by use of genetic markers. Limnol. Oceanogr..

[55] Procheş Ş (2015). Global hotspots in the present-day distribution of ancient animal and plant lineages. Sci. Reports.

[56] Saito T (2018). Molecular phylogeny of glacial relict species: a case of freshwater Valvatidae molluscs (Mollusca: Gastropoda) in North and East Asia. Hydrobiologia.

[57] Ooyagi A (2018). Phylogeography of the eight-barbel loach *Lefua nikkonis* (Cypriniformes: Nemacheilidae): how important were straits in northern Japan as biogeographical barriers?. Ichthyol. Res..

[58] Sawagaki T, Aoki T (2011). Late quaternary glaciations in Japan. Dev. Quat. Sci..

[59] Xiang XF (2015). Check-List of Chinese Cladocera (Crustacea: Branchiopoda). Part 1. Haplopoda, Ctenopoda, Onychopoda and Anomopoda (families Daphniidae, Moinidae, Bosminidae, Ilyocryptidae). Zootaxa.

[60] Korovchinsky NM (2013). Cladocera (Crustacea: Branchiopoda) of South East Asia: history of exploration, taxon richness and notes on zoogeography. J. Limnol..

[61] Kotov AA (2013). Cladocera (Crustacea: Branchiopoda) of Vientiane province and municipality, Laos. J. Limnol..

[62] Sinev AY, Gu Y, Han BP (2015). Cladocera of Hainan Island, China. Zootaxa.

[63] Taira A (2001). Tectonic evolution of the Japanese island arc system. Ann. Rev. Earth Planet. Sci..

[64] Barnes GL (2003). Origins of the Japanese Islands: The New “Big Picture”. Jap. Rev..

[65] Park YC, Kitade O, Schwarz M, Kim JP, Kim W (2006). Intraspecific molecular phylogeny, genetic variation and phylogeography of *Reticulitermes speratus* (Isoptera: Rhinotermitidae). Mol. Cells.

[66] Grandcolas P, Nattier R, Trewick S (2014). Relict species: a relict concept?. TREES.

[67] Grandcolas, P. & Trewick, S. A. What is the meaning of extreme phylogenetic diversity? The case of phylogenetic relict species. In *Biodiversity Conservation and Phylogenetic Systematics* (ed. Pellens, R. & Grandcolas, P.) 99–115 (Springer; 2016).

[68] Urabe J, Ishida S, Nishimoto M, Weider LJ (2003). *Daphnia pulicaria*, a zooplankton species that suddenly appeared in 1999 in the offshore zone of Lake Biwa. Limnology.

[69] Duggan IC, Pullan SG (2017). Do freshwater aquaculture facilities provide an invasion risk for zooplankton hitchhikers?. Biol. Invasions.

[70] Kearse M (2012). Geneious Basic: an integrated and extendable desktop software platform for the organization and analysis of sequence data. Bioinformatics.

[71] Galtier N, Gouy M, Gautier C (1996). SeaView and Phylo_win, two graphic tools for sequence alignment and molecular phylogeny. Comput. Applic. Biosci..

[72] Trifinopoulos J, Nguyen LT, von Haeseler A, Minh BQ (2016). W-IQ-TREE: a fast online phylogenetic tool for maximum likelihood analysis. Nucleic Acids Res..

[73] Nguyen LT, Schmidt HA, von Haeseler A, Minh BQ (2015). IQ-TREE: A fast and effective stochastic algorithm for estimating maximum likelihood phylogenies. Mol. Biol. Evol..

[74] Rambaut, A. FigTree v.1.4: Tree figure drawing tool, http://tree.bio.ed.ac.uk/software/figtree (2008).

[75] Leigh JW, Bryant D (2015). popart: full-feature software for haplotype network construction. Meth. Ecol. Evol..

[76] Murrell B (2013). FUBAR: a fast, unconstrained bayesian approximation for inferring selection. Mol. Biol. Evol..

[77] Delport W, Poon AFY, Frost SDW, Pond SLK (2010). Datamonkey 2010: a suite of phylogenetic analysis tools for evolutionary biology. Bioinformatics.

[78] Rozas J (2017). DnaSP 6: DNA Sequence Polymorphism Analysis of Large Datasets. Mol. Biol. Evol..

