# Supplementary material for: Contrasting endemism in pond-dwelling cyclic parthenogens: the Daphnia curvirostris species group (Crustacea: Cladocera)
Source: Sci Rep. 2019 May 2;9:6812. doi: 10.1038/s41598-019-43281-9 (PMC6497905; doi:10.1038/s41598-019-43281-9)
Supplement: Supplementary file 1 — Supplementary information to the paper: Contrasting endemism in pond-dwelling cyclic parthenogens: the Daphnia curvirostris species group (Crustacea: Cladocera) [file 41598_2019_43281_MOESM1_ESM.pdf]

Supplementary information to:

Contrasting endemism in pond-dwelling cyclic parthenogens: the *Daphnia curvirostris* species group (Crustacea: Cladocera)

Alexey A. Kotov[1*] & Derek J. Taylor[2]

[1]A. N. Severtsov Institute of Ecology and Evolution, Leninsky Prospect 33, Moscow 119071, Russia.

[2] Department of Biological Sciences, The State University of New York at Buffalo, Buffalo, NY 14260, USA.

* Correspondence and requests for material should be addressed to AAK (alexey-a-kotov@yandex.ru)

Supplementary Table S1. Complete list of original sequences (with individual IDs) obtained in this study with information on sampling localities and the GenBank accession numbers for ND2 and HSP sequences for each specimen. ND2 clade designations as defined in Figure 1.

**Clade**	**Taxon name**	**ND2 ID**	**ND2 Genbank number**	**HSP ID**	**HSP Genbank number**	Country	State	Locality	collector	N	E
**A**	***Daphnia curvirostris* s.str.**	**Azerbaijan_1a**	MH614103			Azerbaijan		A large puddle near road, Kyzilagach Reserve	E. V. Bragina	39.1	48.9
**A**	***Daphnia curvirostris* s.str.**	**Azerbaijan_1b**	MH614104			Azerbaijan		A large puddle near road, Kyzilagach Reserve	E. V. Bragina	39.1	48.9
**A**	***Daphnia curvirostris* s.str.**	**Azerbaijan_1c**	MH614168			Azerbaijan		A large puddle near road, Kyzilagach Reserve	E. V. Bragina	39.1	48.9
**A**	***Daphnia curvirostris* s.str.**	**France_1a**	MH614139			France		Nogent, Seine	J. F. Cart	48.50444	3.49389
**A**	***Daphnia curvirostris* s.str.**	**France_1b**	MH614140			France		Nogent, Seine	J. F. Cart	48.50444	3.49389
**A**	***Daphnia curvirostris* s.str.**	**France_1c**	MH614141			France		Nogent, Seine	J. F. Cart	48.50444	3.49389
**A**	***Daphnia curvirostris* s.str.**	**France_1d**	MH613992			France		Nogent, Seine	J. F. Cart	48.50444	3.49389
**A**	***Daphnia curvirostris* s.str.**	**France_1e**	MH614143			France		Nogent, Seine	J. F. Cart	48.50444	3.49389
**A**	***Daphnia curvirostris* s.str.**	**France_1f**	MH614144			France		Nogent, Seine	J. F. Cart	48.50444	3.49389
**A**	***Daphnia curvirostris* s.str.**	**France_1g**	MH614145			France		Nogent, Seine	J. F. Cart	48.50444	3.49389
**A**	***Daphnia curvirostris* s.str.**	**France_2a**	MH614097			France		Alsace, Munchhausen	J. F. Cart	48.913	8.1371
**A**	***Daphnia curvirostris* s.str.**	**France_2b**	MH614098			France		Alsace, Munchhausen	J. F. Cart	48.913	8.1371
**A**	***Daphnia curvirostris* s.str.**	**France_2c**	MH614099			France		Alsace, Munchhausen	J. F. Cart	48.913	8.1371
**A**	***Daphnia curvirostris* s.str.**	**France_2d**	MH614100			France		Alsace, Munchhausen	J. F. Cart	48.913	8.1371
**A**	***Daphnia curvirostris* s.str.**	**France_2e**	MH614126			France		Alsace, Munchhausen	J. F. Cart	48.913	8.1371
**A**	***Daphnia curvirostris* s.str.**	**France_2f**	MH614127			France		Alsace, Munchhausen	J. F. Cart	48.913	8.1371

**A**	***Daphnia curvirostris* s.str.**	**France_2g**	MH614165			France		Alsace, Munchhausen	J. F. Cart	48.913	8.1371
**A**	***Daphnia curvirostris* s.str.**	**France_3a**	MH614115			France		Saron	J. F. Cart	48.5331	3.735
**A**	***Daphnia curvirostris* s.str.**	**France_3b**	MH614116			France		Saron	J. F. Cart	48.5331	3.735
**A**	***Daphnia curvirostris* s.str.**	**France_3c**	MH614120			France		Saron	J. F. Cart	48.5331	3.735
**A**	***Daphnia curvirostris* s.str.**	**France_3d**	MH614121			France		Saron	J. F. Cart	48.5331	3.735
**A**	***Daphnia curvirostris* s.str.**	**France_3e**	MH614150			France		Saron	J. F. Cart	48.5331	3.735
**A**	***Daphnia curvirostris* s.str.**	**France_3f**	MH614164			France		Saron	J. F. Cart	48.5331	3.735
**A**	***Daphnia curvirostris* s.str.**	**France_3g**	MH614166			France		Saron	J. F. Cart	48.5331	3.735
**A**	***Daphnia curvirostris* s.str.**	**France_4a**	MH614107			France		Argeles plage	J. F. Cart	42.57329	3.04447
**A**	***Daphnia curvirostris* s.str.**	**France_4b**	MH614108			France		Argeles plage	J. F. Cart	42.57329	3.04447
**A**	***Daphnia curvirostris* s.str.**	**France_4c**	MH614109			France		Argeles plage	J. F. Cart	42.57329	3.04447
**A**	***Daphnia curvirostris* s.str.**	**France_4d**	MH614110			France		Argeles plage	J. F. Cart	42.57329	3.04447
**A**	***Daphnia curvirostris* s.str.**	**France_4e**	MH614111			France		Argeles plage	J. F. Cart	42.57329	3.04447
**A**	***Daphnia curvirostris* s.str.**	**France_4f**	MH614112			France		Argeles plage	J. F. Cart	42.57329	3.04447
**A**	***Daphnia curvirostris* s.str.**	**France_4g**	MH614113			France		Argeles plage	J. F. Cart	42.57329	3.04447
**A**	***Daphnia curvirostris* s.str.**	**France_4h**	MH614114			France		Argeles plage	J. F. Cart	42.57329	3.04447
**A**	***Daphnia curvirostris* s.str.**	**France_4i**	MH614118			France		Argeles plage	J. F. Cart	42.57329	3.04447
**A**	***Daphnia curvirostris* s.str.**	**France_4j**	MH614119			France		Argeles plage	J. F. Cart	42.57329	3.04447
**A**	***Daphnia curvirostris* s.str.**	**France_5a**	MH614096			France		La prée Seine	J. F. Cart	48.49312	3.50161
**A**	***Daphnia curvirostris* s.str.**	**France_5b**	MH614117			France		La prée Seine	J. F. Cart	48.49312	3.50161
**A**	***Daphnia curvirostris* s.str.**	**France_5c**	MH614132			France		La prée Seine	J. F. Cart	48.49312	3.50161
**A**	***Daphnia curvirostris* s.str.**	**France_5d**	MH614146			France		La prée Seine	J. F. Cart	48.49312	3.50161
**A**	***Daphnia curvirostris* s.str.**	**Georgia_1a**	MH614081	**Georgia_1a**	MH734032	Georgia		well near Bochorma orhodox church	E. V. Popova	41.91	45.15
**A**	***Daphnia curvirostris* s.str.**	**Mexico_1a**	MH614133			Mexico	State of Mexico	Chimaliapan wetlands	S. Nandini		
**A**	***Daphnia curvirostris* s.str.**	**Mexico_1b**	MH614137			Mexico	State of Mexico	Chimaliapan wetlands	S. Nandini		

**A**	***Daphnia curvirostris* s.str.**	**Mongolia_1a**	MH614079	**Mongolia_1a**	MH734036	Mongolia		A large lake near Ungi-Gol River, entrance to the Lake Valley	A. A. Kotov	46.38843	102.7154
**A**	***Daphnia curvirostris* s.str.**	**Mongolia_1b**	MH614083	**Mongolia_1b**	MH734038	Mongolia		A large lake near Ungi-Gol River, entrance to the Lake Valley	A. A. Kotov	46.38843	102.7154
**A**	***Daphnia curvirostris* s.str.**	**Mongolia_1c**	MH614084	**Mongolia_1c**	MH734017	Mongolia		A large lake near Ungi-Gol River, entrance to the Lake Valley	A. A. Kotov	46.38843	102.7154
**A**	***Daphnia curvirostris* s.str.**	**Mongolia_1d**	MH614085	**Mongolia_1d**	MH734028	Mongolia		A large lake near Ungi-Gol River, entrance to the Lake Valley	A. A. Kotov	46.38843	102.7154
**A**	***Daphnia curvirostris* s.str.**	**Mongolia_1e**	MH614086	**Mongolia_1e**	MH734029	Mongolia		A large lake near Ungi-Gol River, entrance to the Lake Valley	A. A. Kotov	46.38843	102.7154
**A**	***Daphnia curvirostris* s.str.**	**Mongolia_1f**	MH614087	**Mongolia_1f**	MH734030	Mongolia		A large lake near Ungi-Gol River, entrance to the Lake Valley	A. A. Kotov	46.38843	102.7154
**A**	***Daphnia curvirostris* s.str.**	**Mongolia_1g**	MH614088	**Mongolia_1g**	MH734031	Mongolia		A large lake near Ungi-Gol River, entrance to the Lake Valley	A. A. Kotov	46.38843	102.7154
**A**	***Daphnia curvirostris* s.str.**	**Mongolia_1h**	MH614091			Mongolia		A large lake near Ungi-Gol River, entrance to the Lake Valley	A. A. Kotov	46.38843	102.7154
**A**	***Daphnia curvirostris* s.str.**	**Mongolia_1i**	MH614092			Mongolia		A large lake near Ungi-Gol River, entrance to the Lake Valley	A. A. Kotov	46.38843	102.7154
**A**	***Daphnia curvirostris* s.str.**	**Mongolia_2a**	MH614094	**Mongolia_2a**	MH734034	Mongolia	Uvs Aimag	A small puddle, left bank of Dzabhan Gol (River)	A. A. Kotov	47.528	95.834
**A**	***Daphnia curvirostris* s.str.**	**Mongolia_2b**	MH614148	**Mongolia_2b**	MH734035	Mongolia	Uvs Aimag	A small puddle, left	A. A. Kotov	47.528	95.834

								bank of Dzabhan Gol (River)			
**A**	***Daphnia curvirostris* s.str.**	**Russia_Volgograd_1a**	MH614093	**Russia_Volgograd_1a**	MH734018	Russia (European)	Volgograd Area	A flooded region near a big lake	Y. R. Galimov	49.97889	46.52861
**A**	***Daphnia curvirostris* s.str.**	**Russia_Karelia_1a**	MH614090	**Russia_Karelia_1a**	MH734026	Russia (European)	Karealian Autonomous Republic	Rock pool, Kirabenavalok Cape, White Sea	D. M. Glazov	65.96318	34.71268
**A**	***Daphnia curvirostris* s.str.**	**Russia_Kurgan_1a**	MH614163			Russia (Asian)	Kurgan Area	A pond, right side of highway M51 near Kumsha	A. A. Kotov	55.31495	63.39903
**A**	***Daphnia curvirostris* s.str.**	**Russia_Kurgan_2a**	MH614162			Russia (Asian)	Kurgan Area	Lake Tchesnochnoje near village of Tchesnokovo (salty)	A. A. Kotov	55.33148	63.48267
**A**	***Daphnia curvirostris* s.str.**	**Russia_Moscow_1a**	MH614082			Russia (European)	Moscow Area	Lake Glubokoe	A. A. Kotov	55.75361	36.50417
**A**	***Daphnia curvirostris* s.str.**	**Russia_Moscow_1b**	MH614134			Russia (European)	Moscow Area	Lake Glubokoe	J. F. Cart	55.75361	36.50417
**A**	***Daphnia curvirostris* s.str.**	**Russia_Moscow_1c**	MH614136			Russia (European)	Moscow Area	Lake Glubokoe	J. F. Cart	55.75361	36.50417
**A**	***Daphnia curvirostris* s.str.**	**Russia_Moscow_1d**	MH614138			Russia (European)	Moscow Area	Lake Glubokoe	J. F. Cart	55.75361	36.50417
**A**	***Daphnia curvirostris* s.str.**	**Russia_Tuva_1a**	MH614169			Russia (Asian)	Tuva	Dorog-Khol lake, Todzha Depression	E.I. Zuykova	52.57	96.26
**A**	***Daphnia curvirostris* s.str.**	**Russia_Volgograd_1b**	MH614089	**Russia_Volgograd_1b**	MH734018	Russia (European)	Volgograd Area	A flooded region near a big lake	Y. R. Galimov	49.97889	46.52861
**A**	***Daphnia curvirostris* s.str.**	**Russia_Yakutia_1a**	MH614105			Russia (Asian)	Yakutia Autonomous Republic	A metallic tank near house of L. Pestriakova, Yakutsk	A. A. Kotov	61.96021	129.6543
**A**	***Daphnia curvirostris* s.str.**	**Russia_Yakutia_1b**	MH614106			Russia (Asian)	Yakutia Autonomous Republic	A metallic tank near house of L. Pestriakova, Yakutsk	A. A. Kotov	61.96021	129.6543
**A**	***Daphnia curvirostris* s.str.**	**Russia_Yakutia_1c**	MH614149			Russia (Asian)	Yakutia Autonomous Republic	A metallic tank near house of L. Pestriakova, Yakutsk	J. F. Cart	61.96021	129.6543
**A**	***Daphnia curvirostris* s.str.**	**Russia_Yakutia_1d**	MH614167			Russia (Asian)	Yakutia Autonomous Republic	A metallic tank near house of L. Pestriakova,	A. A. Kotov	61.96021	129.6543

								Yakutsk			
**A**	***Daphnia curvirostris* s.str.**	**Russia_Yakutia_ 2e**	MH614080	**Russia_Yakutia_2e**	MH734021	Russia (Asian)	Yakutia Autonomous Republic	Lake Tabaga (very shallow)	E.I. Bekker, A.I. Klimovsky	61.829	129.616
**A**	***Daphnia curvirostris* s.str.**	**Russia_Yakutia_ 3a**	MH614075			Russia (Asian)	Yakutia Autonomous Republic	Puddle near settlement of Tuluma	E.I. Bekker, A.I. Klimovsky	62.791	130.056
**A**	***Daphnia curvirostris* s.str.**	**Russia_Yakutia_ 3b**	MH614076			Russia (Asian)	Yakutia Autonomous Republic	Puddle near settlement of Tuluma	E.I. Bekker, A.I. Klimovsky	62.791	130.056
**A**	***Daphnia curvirostris* s.str.**	**Russia_Yakutia_ 3c**	MH614077			Russia (Asian)	Yakutia Autonomous Republic	Puddle near settlement of Tuluma	E.I. Bekker, A.I. Klimovsky	62.791	130.056
**A**	***Daphnia curvirostris* s.str.**	**Russia_Yakutia_ 3d**	MH614078			Russia (Asian)	Yakutia Autonomous Republic	Puddle near settlement of Tuluma	E.I. Bekker, A.I. Klimovsky	62.791	130.056
**A**	***Daphnia curvirostris* s.str.**	**Russia_Yaroslavl _1a**	MH614095			Russia (European)	Yaroslavl Area	Temporary puddles near Borok	A. A. Kotov & A. G. Kirdiasheva	58.06015	38.2169
**A**	***Daphnia curvirostris* s.str.**	**Russia_Yaroslavl _1b**	MH614135			Russia (European)	Yaroslavl Area	Temporary puddles near Borok	A. A. Kotov & A. G. Kirdiasheva	58.06015	38.2169
**A**	***Daphnia curvirostris* s.str.**	**Russia_Yaroslavl _1c**	MH614152			Russia (European)	Yaroslavl Area	Temporary puddles near Borok	A. A. Kotov & A. G. Kirdiasheva	58.06015	38.2169
**A**	***Daphnia curvirostris* s.str.**	**Russia_Yaroslavl _2a**	MH614154			Russia (European)	Yaroslavl Area	Puddles near the "Liagushatnik" , Borok	A. A. Kotov & A. G. Kirdiasheva	58.06453	38.24495
**A**	***Daphnia curvirostris* s.str.**	**Russia_Yaroslavl _2b**	MH614155			Russia (European)	Yaroslavl Area	Puddles near the "Liagushatnik" , Borok	A. A. Kotov & A. G. Kirdiasheva	58.06453	38.24495
**A**	***Daphnia curvirostris* s.str.**	**Russia_Yaroslavl _2c**	MH614156			Russia (European)	Yaroslavl Area	Puddles near the "Liagushatnik" , Borok	A. A. Kotov & A. G. Kirdiasheva	58.06453	38.24495
**A**	***Daphnia curvirostris* s.str.**	**Russia_Yaroslavl _2d**	MH614157			Russia (European)	Yaroslavl Area	Puddles near the "Liagushatnik" , Borok	A. A. Kotov & A. G. Kirdiasheva	58.06453	38.24495
**A**	***Daphnia curvirostris* s.str.**	**Russia_Yaroslavl _2e**	MH614158			Russia (European)	Yaroslavl Area	Puddles near the "Liagushatnik" , Borok	A. A. Kotov & A. G. Kirdiasheva	58.06453	38.24495
**A**	***Daphnia curvirostris* s.str.**	**Russia_Yaroslavl _2f**	MH614159			Russia (European)	Yaroslavl Area	Puddles near the "Liagushatnik" , Borok	A. A. Kotov & A. G. Kirdiasheva	58.06453	38.24495
**A**	***Daphnia curvirostris* s.str.**	**Russia_Yaroslavl _2g**	MH614160			Russia (European)	Yaroslavl Area	Puddles near the "Liagushatnik"	A. A. Kotov & A. G. Kirdiasheva	58.06453	38.24495

								, Borok			
**A**	***Daphnia curvirostris* s.str.**	**Russia_Yaroslavl_2h**	MH614161			Russia (European)	Yaroslavl Area	Puddles near the "Liagushatnik", Borok	A. A. Kotov & A. G. Kirdiasheva	58.06453	38.24495
**A**	***Daphnia curvirostris* s.str.**	**Slovakia_1a**	DQ132620			Slovakia		Somotor (Ishida et al., 2006)		48.4	21.41
**A**	***Daphnia curvirostris* s.str.**	**Ukraine_1a**	MH614101			Ukraine	Odessa Area	A pond near Tiligul River near Beriozovka	E.I. Bekker	47.173	30.9215
**A**	***Daphnia curvirostris* s.str.**	**Ukraine_1b**	MH614129			Ukraine	Odessa Area	A pond near Tiligul River near Beriozovka	E.I. Bekker	47.173	30.9215
**A**	***Daphnia curvirostris* s.str.**	**Ukraine_2a**	MH614102			Ukraine	Odessa Area	A flooded area near Tiligul River near Beriozovka	E.I. Bekker	47.173	30.9214
**A**	***Daphnia curvirostris* s.str.**	**Ukraine_2b**	MH614125			Ukraine	Odessa Area	A flooded area near Tiligul River near Beriozovka	E.I. Bekker	47.173	30.9214
**A**	***Daphnia curvirostris* s.str.**	**Ukraine_2c**	MH614128			Ukraine	Odessa Area	A flooded area near Tiligul River near Beriozovka	E.I. Bekker	47.173	30.9214
**A**	***Daphnia curvirostris* s.str.**	**Ukraine_2d**	MH614130			Ukraine	Odessa Area	A flooded area near Tiligul River near Beriozovka	E.I. Bekker	47.173	30.9214
**A**	***Daphnia curvirostris* s.str.**	**Ukraine_2e**	MH614131			Ukraine	Odessa Area	A flooded area near Tiligul River near Beriozovka	E.I. Bekker	47.173	30.9214
**A**	***Daphnia curvirostris* s.str.**	**Ukraine_2f**	MH614147			Ukraine	Odessa Area	A flooded area near Tiligul River near Beriozovka	E.I. Bekker	47.173	30.9214
**A**	***Daphnia curvirostris* s.str.**	**Ukraine_3a**	MH614151			Ukraine	Lugansk Area	A gees pond near Kuriachje	E.I. Bekker	48.18	39.6
**A**	***Daphnia curvirostris* s.str.**	**Ukraine_3b**	MH614153			Ukraine	Lugansk Area	A gees pond near Kuriachje	D. E. Shcherbakov	48.18	39.6
**A**	***Daphnia curvirostris* s.str.**	**Ukraine_3c**	MH614122			Ukraine	Lugansk Area	A gees pond near Kuriachje	D. E. Shcherbakov	48.18	39.6
**A**	***Daphnia curvirostris* s.str.**	**Ukraine_3d**	MH614123			Ukraine	Lugansk Area	A gees pond near Kuriachje	D. E. Shcherbakov	48.18	39.6
**A**	***Daphnia curvirostris* s.str.**	**Ukraine_3e**	MH614124			Ukraine	Lugansk Area	A gees pond near Kuriachje	D. E. Shcherbakov	48.18	39.6
**A**	***Daphnia curvirostris* s.str.**	**Russia_Volgograd_1c**		**Russia_Volgograd_1c**	MH734025	Russia (European)	Volgograd Area	A flooded region near a	Y. R. Galimov	49.97889	46.52861

								big lake			
**A**	***Daphnia curvirostris* s.str.**	**Georgia_1b**		**Georgia_1b**	MH734033	Georgia		well near Bochorma orhodox church	E. V. Popova	41.91	45.15
**A**	***Daphnia curvirostris* s.str.**			**Russia_Yakutia_2f**	MH734022	Russia (Asian)	Yakutia Autonomous Republic	Lake Tabaga (very shallow)	E.I. Bekker, A.I. Klimovsky	61.829	129.616
**A**	***Daphnia curvirostris* s.str.**			**Russia_Yakutia_2g**	MH734023	Russia (Asian)	Yakutia Autonomous Republic	Lake Tabaga (very shallow)	E.I. Bekker, A.I. Klimovsky	61.829	129.616
**A**	***Daphnia curvirostris* s.str.**			**Russia_Yakutia_2h**	MH734024	Russia (Asian)	Yakutia Autonomous Republic	Lake Tabaga (very shallow)	E.I. Bekker, A.I. Klimovsky	61.829	129.616
**A**	***Daphnia curvirostris* s.str.**			**Russia_Yakutia_2i**	MH734027	Russia (Asian)	Yakutia Autonomous Republic	Lake Tabaga (very shallow)	E.I. Bekker, A.I. Klimovsky	61.829	129.616
**A**	***Daphnia curvirostris* s.str.**			**Mongolia_2c**	MH734037	Mongolia	Uvs Aimag	A small puddle, left bank of Dzabhan Gol (River)	A. A. Kotov	47.528	95.834
**B**	***Daphnia* cf. *curvirostris***	**Russia_Khabarovsk_3b**	MH614068			Russia (Asian)	Khabarovsk Territory	Puddle 2, Pionerskaya Street, region of Piataya Lodochnaya Stantsiya, town of Khabarovsk	A. A. Kotov & N. M. Korovchinsky	48.39466	135.0907
**B**	***Daphnia* cf. *curvirostris***	**Russia_Khabarovsk_4a**	MH614074			Russia (Asian)	Khabarovsk Territory	A roadside ditch, Pionerskaya Street, town of Khabarovsk	A. A. Kotov & N. M. Korovchinsky	48.40395	135.0989
**B**	***Daphnia* cf. *curvirostris***	**Russia_Khabarovsk_7a**	MH614065	**Russia_Khabarovsk_7a**	MH734010	Russia (Asian)	Khabarovsk Territory	A puddle near the bridge across the River Aniuy, left side of the highway Khabarovsk - Komsomol'sk-na-Amure	A. A. Kotov & N. M. Korovchinsky	49.30617	136.5052
**B**	***Daphnia* cf. *curvirostris***	**Russia_Khabarovsk_7b**	MH614067	**Russia_Khabarovsk_7b**	MH734013	Russia (Asian)	Khabarovsk Territory	A puddle near the bridge across the River Aniuy, left side of the highway Khabarovsk - Komsomol'sk-	A. A. Kotov & N. M. Korovchinsky	49.30617	136.5052

								na-Amure			
**B**	***Daphnia* cf. *curvirostris***	**Russia_Primorski_5l**	MH614063			Russia (Asian)	Primorski Territory	Puddle 1 near Komarovka River, Ussurisky Nature Reserve	P. A. Sorokin	43.64833	132.3951
**B**	***Daphnia* cf. *curvirostris***	**Russia_Primorski_5m**	MH614064			Russia (Asian)	Primorski Territory	Puddle 1 near Komarovka River, Ussurisky Nature Reserve	P. A. Sorokin	43.64833	132.3951
**B**	***Daphnia* cf. *curvirostris***	**Russia_Primorski_5n**	MH614073			Russia (Asian)	Primorski Territory	Puddle 1 near Komarovka River, Ussurisky Nature Reserve	P. A. Sorokin	43.64833	132.3951
**B**	***Daphnia* cf. *curvirostris***	**Russia_Sakhalin_1b**	MH614066	**Russia_Sakhalin_1b**	MH734014	Russia (Asian)	Sakhalin Area	Puddle 1 in the City Park, Tymovskoe	A. A. Kotov & N. M. Korovchinsky	50.85539	142.655
**B**	***Daphnia* cf. *curvirostris***	**Russia_Yakutia_2a**	MH614069	**Russia_Yakutia_2a**	MH734015	Russia (Asian)	Yakutia Autonomous Republic	Lake Tabaga (very shallow)	E.I. Bekker, A.I. Klimovsky	61.829	129.616
**B**	***Daphnia* cf. *curvirostris***	**Russia_Yakutia_2b**	MH614070	**Russia_Yakutia_2b**	MH734016	Russia (Asian)	Yakutia Autonomous Republic	Lake Tabaga (very shallow)	E.I. Bekker, A.I. Klimovsky	61.829	129.616
**B**	***Daphnia* cf. *curvirostris***	**Russia_Yakutia_2c**	MH614071	**Russia_Yakutia_2c**	MH734019	Russia (Asian)	Yakutia Autonomous Republic	Lake Tabaga (very shallow)	E.I. Bekker, A.I. Klimovsky	61.829	129.616
**B**	***Daphnia* cf. *curvirostris***	**Russia_Yakutia_2d**	MH614072	**Russia_Yakutia_2d**	MH734020	Russia (Asian)	Yakutia Autonomous Republic	Lake Tabaga (very shallow)	E.I. Bekker, A.I. Klimovsky	61.829	129.616
**C**	***Daphnia* cf. *curvirostris***	**USA_Alaska_1a**		**DQ845255**	DQ845255	U.S.A.	Alaska	Pilgrim hotsprings	D. J. Taylor	65.08895	-164.9228
**D**	***Daphnia hrbaceki***	**Czechia_1a**	HM625749			Czechia		Cesky prikop near Nosalov (Jurcka et al., 2010)		50.4817	14.6861
**E**	***Daphnia tanakai***	**Japan_1a**	DQ132618	**DQ845254**	DQ845254	Japan	Honshu Island	Midori-ga-ike, Toyama	S. Tanaka	36.58333	137.597
**E**	***Daphnia tanakai***	**Japan_1b**	DQ132617			Japan	Honshu Island	Midori-ga-ike, Toyama	S. Tanaka	36.58333	137.597
**E**	***Daphnia tanakai***	**Japan_1c**	DQ132616			Japan	Honshu Island	Midori-ga-ike, Toyama	S. Tanaka	36.58333	137.597
**E**	***Daphnia tanakai***	**Japan_3a**		**DQ845253**	DQ845253	Japan	Honshu Island	Kagami-Ike, Gifu		35.41	136.91
**F**	***Daphnia* cf. *sinevi***	**Russia_Sakhalin_1a**	MH614026	**Russia_Sakhalin_1a**	MH734012	Russia (Asian)	Sakhalin Area	Puddle 1 in the City Park, Tymovskoe	A. A. Kotov & N. M. Korovchinsky	50.85539	142.655

**F**	***Daphnia* cf. *sinevi***	**Russia_Sakhalin_2a**	MH614014	**Russia_Sakhalin_2a**	MH733963	Russia (Asian)	Sakhalin Area	Japanese sewage pond 3	A. A. Kotov & N. M. Korovchinsky	47.31561	142.7009
**F**	***Daphnia* cf. *sinevi***	**Russia_Sakhalin_2b**	MH614015	**Russia_Sakhalin_2b**	MH733965	Russia (Asian)	Sakhalin Area	Japanese sewage pond 3	A. A. Kotov & N. M. Korovchinsky	47.31561	142.7009
**F**	***Daphnia* cf. *sinevi***	**Russia_Sakhalin_2c**	MH614016	**Russia_Sakhalin_2c**	MH733966	Russia (Asian)	Sakhalin Area	Japanese sewage pond 3	A. A. Kotov & N. M. Korovchinsky	47.31561	142.7009
**F**	***Daphnia* cf. *sinevi***	**Russia_Sakhalin_2d**	MH614017	**Russia_Sakhalin_2d**	MH733968	Russia (Asian)	Sakhalin Area	Japanese sewage pond 3	A. A. Kotov & N. M. Korovchinsky	47.31561	142.7009
**F**	***Daphnia* cf. *sinevi***	**Russia_Sakhalin_2e**	MH614018	**Russia_Sakhalin_2e**	MH733969	Russia (Asian)	Sakhalin Area	Japanese sewage pond 3	A. A. Kotov & N. M. Korovchinsky	47.31561	142.7009
**F**	***Daphnia* cf. *sinevi***	**Russia_Sakhalin_2f**	MH614019	**Russia_Sakhalin_2f**	MH733973	Russia (Asian)	Sakhalin Area	Japanese sewage pond 3	A. A. Kotov & N. M. Korovchinsky	47.31561	142.7009
**F**	***Daphnia* cf. *sinevi***	**Russia_Sakhalin_2g**	MH614020	**Russia_Sakhalin_2g**	MH733974	Russia (Asian)	Sakhalin Area	Japanese sewage pond 3	A. A. Kotov & N. M. Korovchinsky	47.31561	142.7009
**F**	***Daphnia* cf. *sinevi***	**Russia_Sakhalin_2h**	MH614029	**Russia_Sakhalin_2h**	MH733977	Russia (Asian)	Sakhalin Area	Japanese sewage pond 3	A. A. Kotov & N. M. Korovchinsky	47.31561	142.7009
**F**	***Daphnia* cf. *sinevi***	**Russia_Sakhalin_3a**	MH614021			Russia (Asian)	Sakhalin Area	Japanese sewage pond 3	A. A. Kotov & N. M. Korovchinsky	47.31561	142.7009
**F**	***Daphnia* cf. *sinevi***	**Russia_Sakhalin_3b**	MH614022			Russia (Asian)	Sakhalin Area	Japanese sewage pond 3	A. A. Kotov & N. M. Korovchinsky	47.31561	142.7009
**F**	***Daphnia* cf. *sinevi***	**Russia_Sakhalin_3c**	MH614023			Russia (Asian)	Sakhalin Area	Japanese sewage pond 3	A. A. Kotov & N. M. Korovchinsky	47.31561	142.7009
**F**	***Daphnia* cf. *sinevi***	**Russia_Sakhalin_3d**	MH614030			Russia (Asian)	Sakhalin Area	Japanese sewage pond 3	A. A. Kotov & N. M. Korovchinsky	47.31561	142.7009
**F**	***Daphnia* cf. *sinevi***	**Russia_Sakhalin_5a**	MH614024			Russia (Asian)	Sakhalin Area	Japanese sewage pond 3	A. A. Kotov & N. M. Korovchinsky	47.31561	142.7009
**F**	***Daphnia* cf. *sinevi***	**Russia_Sakhalin_6a**	MH614025	**Russia_Sakhalin_6a**	MH733970	Russia (Asian)	Sakhalin Area	A small forest pool near "Japanese" seawage ponds	A. A. Kotov & N. M. Korovchinsky	47.31642	142.7005
**F**	***Daphnia* cf. *sinevi***	**Russia_Sakhalin_7a**	MH614027	**Russia_Sakhalin_7a**	MH733972	Russia (Asian)	Sakhalin Area	Remains of dried pond 2 near Japanese seawage ponds	A. A. Kotov & N. M. Korovchinsky	47.31728	142.6994
**F**	***Daphnia* cf. *sinevi***	**Russia_Sakhalin_7b**	MH614028	**Russia_Sakhalin_7b**	MH733976	Russia (Asian)	Sakhalin Area	Remains of dried pond 2 near Japanese seawage ponds	A. A. Kotov & N. M. Korovchinsky	47.31728	142.6994
**F**	***Daphnia* cf. *sinevi***			**Russia_Sakhalin_7c**	MH733971	Russia (Asian)	Sakhalin Area	Remains of dried pond 2 near Japanese seawage ponds	A. A. Kotov & N. M. Korovchinsky	47.31728	142.6994
**G**	***Daphnia* cf. *sinevi***	**Russia_Primorski_5a**	MH614034	**Russia_Primorski_5a**	MH733954	Russia (Asian)	Primorski Territory	Puddle 1 near Komarovka River, Ussurisky Nature Reserve	P. A. Sorokin	43.64833	132.3951
**G**	***Daphnia* cf. *sinevi***	**Russia_Primorski_5b**	MH614035	**Russia_Primorski_5b**	MH733955	Russia (Asian)	Primorski Territory	Puddle 1 near Komarovka River, Ussurisky	P. A. Sorokin	43.64833	132.3951

								Nature Reserve			
**G**	***Daphnia* cf. *sinevi***	**Russia_Primorski_5c**	MH614036	**Russia_Primorski_5c**	MH733957	Russia (Asian)	Primorski Territory	Puddle 1 near Komarovka River, Ussurisky Nature Reserve	P. A. Sorokin	43.64833	132.3951
**G**	***Daphnia* cf. *sinevi***	**Russia_Primorski_5d**	MH614037	**Russia_Primorski_5d**	MH733979	Russia (Asian)	Primorski Territory	Puddle 1 near Komarovka River, Ussurisky Nature Reserve	P. A. Sorokin	43.64833	132.3951
**G**	***Daphnia* cf. *sinevi***	**Russia_Primorski_5i**	MH614032			Russia (Asian)	Primorski Territory	Puddle 1 near Komarovka River, Ussurisky Nature Reserve	P. A. Sorokin	43.64833	132.3951
**G**	***Daphnia* cf. *sinevi***	**Russia_Primorski_5j**	MH614031			Russia (Asian)	Primorski Territory	Puddle 1 near Komarovka River, Ussurisky Nature Reserve	P. A. Sorokin	43.64833	132.3951
**G**	***Daphnia* cf. *sinevi***	**Russia_Primorski_5k**	MH614033			Russia (Asian)	Primorski Territory	Puddle 1 near Komarovka River, Ussurisky Nature Reserve	P. A. Sorokin	43.64833	132.3951
**H**	***Daphnia sinevi* s.str.**	**Russia_Khabarovsk_1a**	MH614046			Russia (Asian)	Khabarovsk Territory	A roadside ditch, road to village of Sinda	A. A. Kotov & N. M. Korovchinsky	48.97978	136.3089
**H**	***Daphnia sinevi* s.str.**	**Russia_Khabarovsk_1b**	MH614061			Russia (Asian)	Khabarovsk Territory	A roadside ditch, road to village of Sinda	A. A. Kotov & N. M. Korovchinsky	48.97978	136.3089
**H**	***Daphnia sinevi* s.str.**	**Russia_Khabarovsk_2a**	MH614038	**Russia_Khabarovsk_2a**	MH733953	Russia (Asian)	Khabarovsk Territory	Puddle 1, Pionerskaya Street, region of Piataya Lodochnaya Stantsiya, town of Khabarovsk	A. A. Kotov & N. M. Korovchinsky	48.39455	135.0905
**H**	***Daphnia sinevi* s.str.**	**Russia_Khabarovsk_2b**	MH614039	**Russia_Khabarovsk_2b**	MH733958	Russia (Asian)	Khabarovsk Territory	Puddle 1, Pionerskaya Street, region of Piataya	A. A. Kotov & N. M. Korovchinsky	48.39455	135.0905

								Lodochnaya Stantsiya, town of Khabarovsk			
**H**	***Daphnia sinevi* s.str.**	**Russia_Khabarovsk_2c**	MH614040	**Russia_Khabarovsk_2c**	MH733960	Russia (Asian)	Khabarovsk Territory	Puddle 1, Pionerskaya Street, region of Piataya Lodochnaya Stantsiya, town of Khabarovsk	A. A. Kotov & N. M. Korovchinsky	48.39455	135.0905
**H**	***Daphnia sinevi* s.str.**	**Russia_Khabarovsk_2d**	MH614041	**Russia_Khabarovsk_2d**	MH733961	Russia (Asian)	Khabarovsk Territory	Puddle 1, Pionerskaya Street, region of Piataya Lodochnaya Stantsiya, town of Khabarovsk	A. A. Kotov & N. M. Korovchinsky	48.39455	135.0905
**H**	***Daphnia sinevi* s.str.**	**Russia_Khabarovsk_2e**	MH614044	**Russia_Khabarovsk_2e**	MH733962	Russia (Asian)	Khabarovsk Territory	Puddle 1, Pionerskaya Street, region of Piataya Lodochnaya Stantsiya, town of Khabarovsk	A. A. Kotov & N. M. Korovchinsky	48.39455	135.0905
**H**	***Daphnia sinevi* s.str.**	**Russia_Khabarovsk_2f**	MH614047	**Russia_Khabarovsk_2f**	MH733975	Russia (Asian)	Khabarovsk Territory	Puddle 1, Pionerskaya Street, region of Piataya Lodochnaya Stantsiya, town of Khabarovsk	A. A. Kotov & N. M. Korovchinsky	48.39455	135.0905
**H**	***Daphnia sinevi* s.str.**	**Russia_Khabarovsk_3a**	MH614048	**Russia_Khabarovsk_3a**	MH733980	Russia (Asian)	Khabarovsk Territory	Puddle 2, Pionerskaya Street, region of Piataya Lodochnaya Stantsiya, town of Khabarovsk	A. A. Kotov & N. M. Korovchinsky	48.39466	135.0907
**H**	***Daphnia sinevi* s.str.**	**Russia_Khabarovsk_3c**	MH614045			Russia (Asian)	Khabarovsk Territory	Puddle 2, Pionerskaya Street, region of Piataya Lodochnaya Stantsiya, town of Khabarovsk	A. A. Kotov & N. M. Korovchinsky	48.39466	135.0907

**H**	***Daphnia sinevi*** **s.str.**	**Russia_Khabarovsk_8a**	MH614057			Russia (Asian)	Khabarovsk Territory	Puddle 1, Pionerskaya Street, region of Piataya Lodochnaya Stantsiya, town of Khabarovsk	S. A. Ivanov	48.39455	135.0905
**H**	***Daphnia sinevi*** **s.str.**	**Russia_Primorski_1a**	MH614059	**DQ845251.1**	DQ845251.1	Russia (Asian)	Primorski Territory	A pond 10 m in diameter in Avangard, Nakhodka Area	A. Y. Sinev	42.89	132.72
**H**	***Daphnia sinevi*** **s.str.**	**Russia_Primorski_2a**	MH614049	**Russia_Primorski_2a**	MH733978	Russia (Asian)	Primorski Territory	Puddle 2 near the reservoir of Luchegorskaya a power station	A. A. Kotov & S. A. Ivanov	46.45208	134.2991
**H**	***Daphnia sinevi*** **s.str.**	**Russia_Primorski_2b**	MH614051			Russia (Asian)	Primorski Territory	Puddle 2 near the reservoir of Luchegorskaya a power station	A. A. Kotov & S. A. Ivanov	46.45208	134.2991
**H**	***Daphnia sinevi*** **s.str.**	**Russia_Primorski_3a**	MH614058	**Russia_Primorski_3a**	MH733956	Russia (Asian)	Primorski Territory	A puddle, Partizanskaya Street 1, village of Fedoseevka	A. A. Kotov & S. A. Ivanov	46.53117	134.2777
**H**	***Daphnia sinevi*** **s.str.**	**Russia_Primorski_4a**	MH614060			Russia (Asian)	Primorski Territory	A puddle near the shore of Khanka Lake near the TINRO station, State Khankaisky Biosphere Reserve	N. M. Korovchinsky	44.76017	132.0577
**H**	***Daphnia sinevi*** **s.str.**	**Russia_Primorski_4b**	MH614062			Russia (Asian)	Primorski Territory	A puddle near the shore of Khanka Lake near the TINRO station, State Khankaisky Biosphere Reserve	N. M. Korovchinsky	44.76017	132.0577
**H**	***Daphnia sinevi*** **s.str.**	**Russia_Primorski_5e**	MH614042	**Russia_Primorski_5e**	MH733959	Russia (Asian)	Primorski Territory	Puddle 1 near Komarovka River, Ussurisky	P. A. Sorokin	43.64833	132.3951

								Nature Reserve			
**H**	***Daphnia sinevi* s.str.**	**Russia_Primorski_5f**	MH614043			Russia (Asian)	Primorski Territory	Puddle 1 near Komarovka River, Ussurisky Nature Reserve	P. A. Sorokin	43.64833	132.3951
**H**	***Daphnia sinevi* s.str.**	**Russia_Primorski_5g**	MH614055			Russia (Asian)	Primorski Territory	Puddle 1 near Komarovka River, Ussurisky Nature Reserve	P. A. Sorokin	43.64833	132.3951
**H**	***Daphnia sinevi* s.str.**	**Russia_Primorski_5h**	MH614056			Russia (Asian)	Primorski Territory	Puddle 1 near Komarovka River, Ussurisky Nature Reserve	P. A. Sorokin	43.64833	132.3951
**H**	***Daphnia sinevi* s.str.**	**Russia_Primorski_6a**	MH614050			Russia (Asian)	Primorski Territory	Puddle 2 near Komarovka River, Ussurisky Nature Reserve	P. A. Sorokin	43.64835	132.3951
**H**	***Daphnia sinevi* s.str.**	**Russia_Primorski_6b**	MH614052			Russia (Asian)	Primorski Territory	Puddle 2 near Komarovka River, Ussurisky Nature Reserve	P. A. Sorokin	43.64835	132.3951
**H**	***Daphnia sinevi* s.str.**	**Russia_Primorski_6c**	MH614053			Russia (Asian)	Primorski Territory	Puddle 2 near Komarovka River, Ussurisky Nature Reserve	P. A. Sorokin	43.64835	132.3951
**H**	***Daphnia sinevi* s.str.**	**Russia_Primorski_6d**	MH614054			Russia (Asian)	Primorski Territory	Puddle 2 near Komarovka River, Ussurisky Nature Reserve	P. A. Sorokin	43.64835	132.3951
**I**	***Daphnia* sp. nov.**	**Russia_Khabarovsk_5a**	MH614007	**Russia_Khabarovsk_5a**	MH734005	Russia (Asian)	Khabarovsk Territory	A pond near Vinogradovka village, near a road to Khohlatskaja Protoka of the Amur	A. A. Kotov & N. M. Korovchinsky	48.62314	135.1366

**I**	***Daphnia* sp. nov.**	**Russia_Khabarovsk_5b**	MH614008	**Russia_Khabarovsk_5b**	MH734006	Russia (Asian)	Khabarovsk Territory	A pond near Vinogradovka village, near a road to Khohlatskaja Protoka of the Amur	A. A. Kotov & N. M. Korovchinsky	48.62314	135.1366
**I**	***Daphnia* sp. nov.**	**Russia_Khabarovsk_5c**	MH614009			Russia (Asian)	Khabarovsk Territory	A pond near Vinogradovka village, near a road to Khohlatskaja Protoka of the Amur	A. A. Kotov & N. M. Korovchinsky	48.62314	135.1366
**I**	***Daphnia* sp. nov.**	**Russia_Khabarovsk_5d**	MH614011			Russia (Asian)	Khabarovsk Territory	A pond near Vinogradovka village, near a road to Khohlatskaja Protoka of the Amur	A. A. Kotov & N. M. Korovchinsky	48.62314	135.1366
**I**	***Daphnia* sp. nov.**	**Russia_Khabarovsk_6a**	MH614010			Russia (Asian)	Khabarovsk Territory	A pond near Vinogradovka village, near a road to Khohlatskaja Protoka of the Amur	A. A. Kotov & N. M. Korovchinsky	48.62314	135.1366
**J**	***Daphnia* sp. nov.**	**Japan_2a**	MH614012	**DQ845252**	DQ845252	Japan	Honshu Island	Ootori Ike, Tsuru-Oka City, Yamagata Prefecture	S Ishida	38.3658	139.8302
**J**	***Daphnia* sp. nov.**	**Japan_2b**	MH614013			Japan	Honshu Island	Ootori Ike, Tsuru-Oka City, Yamagata Prefecture	S Ishida	38.3658	139.8302
**K**	***Daphnia* sp. nov.**	**SouthKorea_Jeju_1a**	MH613996	**SouthKorea_Jeju_1a**	MH733981	South Korea	Jeju-do	DoSun-cheon pool 1	A.A. Kotov & H.G. Jeong	33.30593	126.4672
**K**	***Daphnia* sp. nov.**	**SouthKorea_Jeju_1b**	MH613999	**SouthKorea_Jeju_1b**	MH733982	South Korea	Jeju-do	DoSun-cheon pool 1	A.A. Kotov & H.G. Jeong	33.30593	126.4672
**K**	***Daphnia* sp. nov.**	**SouthKorea_Jeju_1c**	MH614000	**SouthKorea_Jeju_1c**	MH733983	South Korea	Jeju-do	DoSun-cheon pool 1	A.A. Kotov & H.G. Jeong	33.30593	126.4672
**K**	***Daphnia* sp. nov.**	**SouthKorea_Jeju_2a**	MH614001	**SouthKorea_Jeju_2a**	MH734002	South Korea	Jeju-do	DoSun-cheon pool 2	A.A. Kotov & H.G. Jeong	33.30595	126.4658
**K**	***Daphnia* sp. nov.**	**SouthKorea_Jeju_2b**	MH613997	**SouthKorea_Jeju_2b**	MH734007	South Korea	Jeju-do	DoSun-cheon pool 2	A.A. Kotov & H.G. Jeong	33.30595	126.4658
**K**	***Daphnia* sp. nov.**	**SouthKorea_Jeju_3a**	MH614004	**SouthKorea_Jeju_3a**	MH733987	South Korea	Jeju-do	pond1 near Hallasan	A.A. Kotov & H.G. Jeong	33.43225	126.5983
**K**	***Daphnia* sp. nov.**	**SouthKorea_Jeju_4a**	MH614003	**SouthKorea_Jeju_4a**	MH733984	South Korea	Jeju-do	Pond near Dream Forest	A.A. Kotov & H.G. Jeong	33.48719	126.7031
**K**	***Daphnia* sp. nov.**	**SouthKorea_Jeju_4b**	MH614006	**SouthKorea_Jeju_4b**	MH733985	South Korea	Jeju-do	Pond near Dream Forest	H.G. Jeong	33.48719	126.7031

**K**	***Daphnia* sp. nov.**	**SouthKorea_Jeju _4c**	MH613998	**SouthKorea_Jeju_4c**	MH733986	South Korea	Jeju-do	Pond near Dream Forest	A.A. Kotov & H.G. Jeong	33.48719	126.7031
**K**	***Daphnia* sp. nov.**	**SouthKorea_Jeju _4e**	MH614002			South Korea	Jeju-do	Pond near Dream Forest	A.A. Kotov & H.G. Jeong	33.48719	126.7031
**K**	***Daphnia* sp. nov.**	**SouthKorea_Jeju _4f**	MH614005	**SouthKorea_Jeju_4f**	MH733992	South Korea	Jeju-do	Pond near Dream Forest	A.A. Kotov & H.G. Jeong	33.48719	126.7031
**K**	***Daphnia* sp. nov.**			**SouthKorea_Jeju_3b**	MH733989	South Korea	Jeju-do	pond1 near Hallasan	A.A. Kotov & H.G. Jeong	33.43225	126.5983
**K**	***Daphnia* sp. nov.**			**SouthKorea_Jeju_3c**	MH733990	South Korea	Jeju-do	pond1 near Hallasan	A.A. Kotov & H.G. Jeong	33.43225	126.5983
**K**	***Daphnia* sp. nov.**			**SouthKorea_Jeju_3d**	MH733991	South Korea	Jeju-do	pond1 near Hallasan	A.A. Kotov & H.G. Jeong	33.43225	126.5983
**K**	***Daphnia* sp. nov.**			**SouthKorea_Jeju_1d**	MH733993	South Korea	Jeju-do	DoSun-cheon pool 1	A.A. Kotov & H.G. Jeong	33.30593	126.4672
**K**	***Daphnia* sp. nov.**			**SouthKorea_Jeju_1f**	MH733995	South Korea	Jeju-do	DoSun-cheon pool 1	A.A. Kotov & H.G. Jeong	33.30593	126.4672
**K**	***Daphnia* sp. nov.**			**SouthKorea_Jeju_1g**	MH733996	South Korea	Jeju-do	DoSun-cheon pool 1	A.A. Kotov & H.G. Jeong	33.30593	126.4672
**K**	***Daphnia* sp. nov.**			**SouthKorea_Jeju_1h**	MH734003	South Korea	Jeju-do	DoSun-cheon pool 1	A.A. Kotov & H.G. Jeong	33.30593	126.4672
**K**	***Daphnia* sp. nov.**			**SouthKorea_Jeju_1i**	MH734004	South Korea	Jeju-do	DoSun-cheon pool 1	A.A. Kotov & H.G. Jeong	33.30593	126.4672
**L**	***Daphnia* sp. nov.**	**SouthKorea_1a**	MH613987	**SouthKorea_1a**	MH733994	South Korea	Chungcheongnam -do	Shallow pool near Deo Seong auditorium	H.G. Jeong, A.A. Kotov, M.A. Gololobova, M.A. Kotova	36.51391	127.2669
**L**	***Daphnia* sp. nov.**	**SouthKorea_1b**	MH613988	**SouthKorea_1b**	MH733997	South Korea	Chungcheongnam -do	Shallow pool near Deo Seong auditorium	H.G. Jeong, A.A. Kotov, M.A. Gololobova, M.A. Kotova	36.51391	127.2669
**L**	***Daphnia* sp. nov.**	**SouthKorea_1c**	MH613989	**SouthKorea_1c**	MH733998	South Korea	Chungcheongnam -do	Shallow pool near Deo Seong auditorium	H.G. Jeong, A.A. Kotov, M.A. Gololobova, M.A. Kotova	36.51391	127.2669
**L**	***Daphnia* sp. nov.**	**SouthKorea_1d**	MH613990	**SouthKorea_1d**	MH733999	South Korea	Chungcheongnam -do	Shallow pool near Deo Seong auditorium	H.G. Jeong, A.A. Kotov, M.A. Gololobova, M.A. Kotova	36.51391	127.2669
**L**	***Daphnia* sp. nov.**	**SouthKorea_1e**	MH613991	**SouthKorea_1e**	MH734000	South Korea	Chungcheongnam -do	Shallow pool near Deo Seong auditorium	H.G. Jeong, A.A. Kotov, M.A. Gololobova, M.A. Kotova	36.51391	127.2669
**L**	***Daphnia* sp. nov.**	**SouthKorea_1f**	MH613992	**SouthKorea_1f**	MH734001	South Korea	Chungcheongnam -do	Shallow pool near Deo Seong auditorium	H.G. Jeong, A.A. Kotov, M.A. Gololobova, M.A. Kotova	36.51391	127.2669
**L**	***Daphnia* sp. nov.**	**SouthKorea_1g**	MH613993			South Korea	Chungcheongnam -do	Shallow pool near Deo Seong auditorium	H.G. Jeong, A.A. Kotov, M.A. Gololobova, M.A. Kotova	36.51391	127.2669
**L**	***Daphnia* sp. nov.**	**SouthKorea_1h**	MH613994			South Korea	Chungcheongnam -do	Shallow pool near Deo	H.G. Jeong, A.A. Kotov, M.A. Gololobova, M.A.	36.51391	127.2669

								Seong auditorium	Kotova		
**L**	***Daphnia* sp. nov.**	**SouthKorea_1i**	MH613995			South Korea	Chungcheongnam-do	Shallow pool near Deo Seong auditorium	H.G. Jeong, A.A. Kotov, M.A. Gololobova, M.A. Kotova	36.51391	127.2669
	Daphnia_cristata	**Daphnia cristata**		**DQ845262**	DQ845262						
	Daphnia_longiremis	**Daphnia longiremis**		**DQ845263**	DQ845263						
	Daphnia_dubia	**Daphnia dubia**		**DQ845261**	DQ845261						
	Daphnia_laevis	**Daphnia laevis**		**DQ845260**	DQ845260						
	Daphnia galeata	**Daphnia galeata**		**ABJ16337.1**							
	Daphnia unbra	**Daphnia unbra**		**ABA02317.1**							

Supplementary Figure S1. Maximum likelihood tree based on sequences of the mitochondrial *ND2* gene representing the diversity among phylogroups of the *Daphnia curvirostris* group with members of *D.laevis-D.longiremis* group as a root. The support values of individual nodes are based on maximum likelihood. Colors and shapes for main phylogroups correspond to those in Fig. 1.

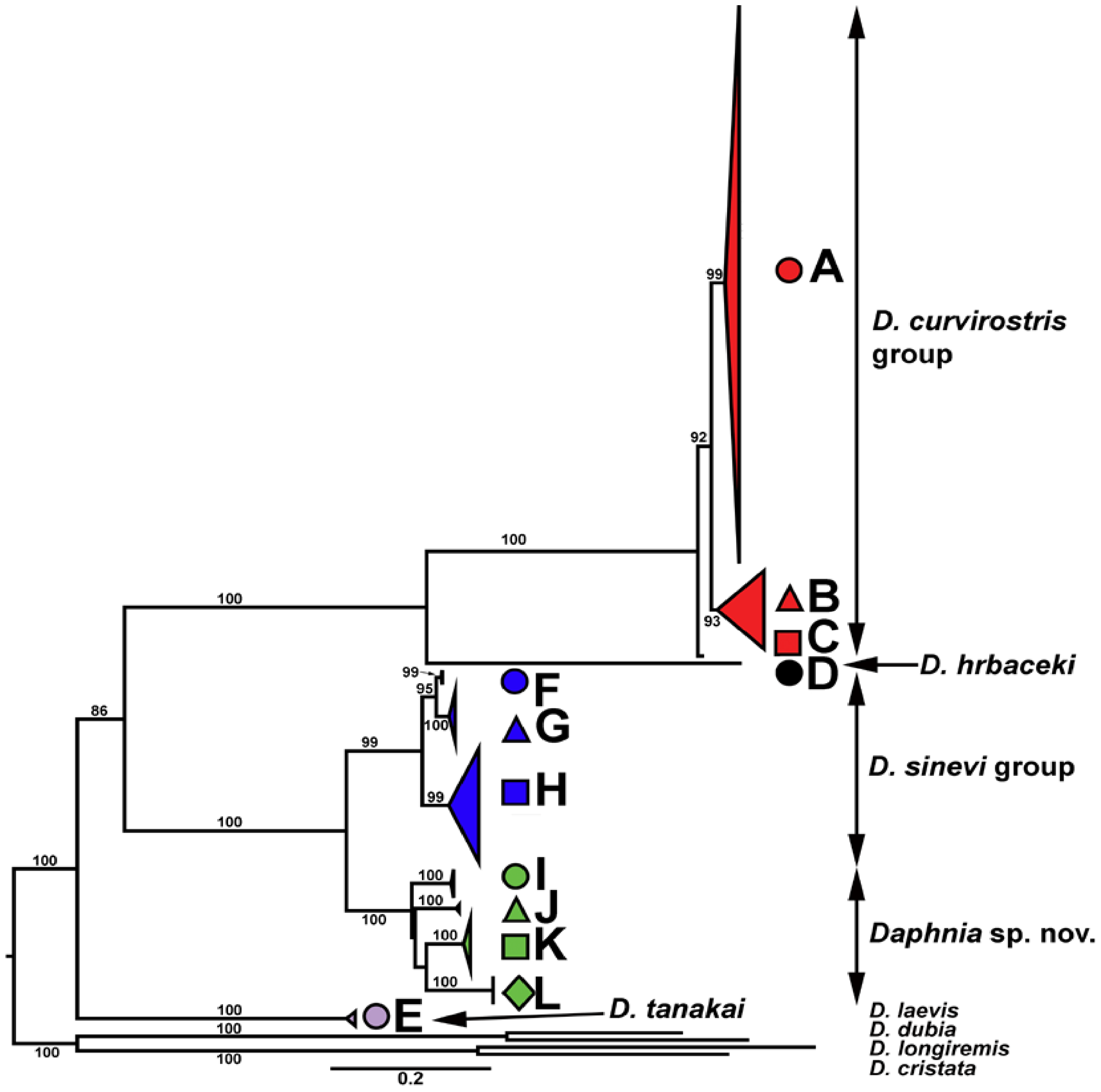

Supplementary Figure S2. Maximum Likelihood phylogram of the ND2 region from the *Daphnia curvirostris* complex based on an amino acid alignment and the mtart (arthropod-specific) substitution model with members of *D.laevis-D.longiremis* group as a root. Support values are given on the branches (Approximate LRT's). Colors represent major clades as given in Figure1.

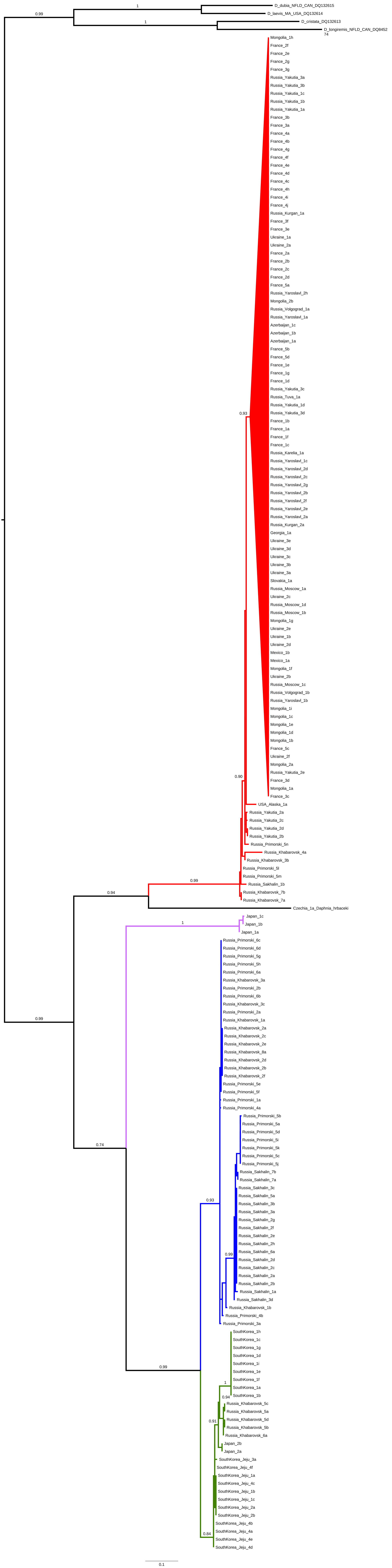
D_dubia_NFLD_CAN_DQ132615
D_laevis_MA_USA_DQ132614
D_cristata_DQ132613
D_longiremis_NFLD_CAN_DQ845274
Mongolia_1h
France_2f
France_2e
France_2g
France_3g
Russia_Yakutia_3a
Russia_Yakutia_3b
Russia_Yakutia_1c
Russia_Yakutia_1b
Russia_Yakutia_1a
France_3b
France_3a
France_4a
France_4b
France_4g
France_4f
France_4e
France_4d
France_4c
France_4h
France_4i
France_4j
Russia_Kurgan_1a
France_3f
France_3e
Ukraine_1a
Ukraine_2a
France_2a
France_2b
France_2c
France_2d
France_5a
Russia_Yaroslavl_2h
Mongolia_2b
Russia_Volgograd_1a
Russia_Yaroslavl_1a
Azerbaijan_1c
Azerbaijan_1b
Azerbaijan_1a
France_5b
France_5d
France_1e
France_1g
France_1d
Russia_Yakutia_3c
Russia_Tuva_1a
Russia_Yakutia_1d
Russia_Yakutia_3d
France_1b
France_1a
France_1f
France_1c
Russia_Karelia_1a
Russia_Yaroslavl_1c
Russia_Yaroslavl_2d
Russia_Yaroslavl_2c
Russia_Yaroslavl_2g
Russia_Yaroslavl_2b
Russia_Yaroslavl_2f
Russia_Yaroslavl_2e
Russia_Yaroslavl_2a
Russia_Kurgan_2a
Georgia_1a
Ukraine_3e
Ukraine_3d
Ukraine_3c
Ukraine_3b
Ukraine_3a
Slovakia_1a
Russia_Moscow_1a
Ukraine_2c
Russia_Moscow_1d
Russia_Moscow_1b
Mongolia_1g
Ukraine_2e
Ukraine_1b
Ukraine_2d
Mexico_1b
Mexico_1a
Mongolia_1f
Ukraine_2b
Russia_Moscow_1c
Russia_Volgograd_1b
Russia_Yaroslavl_1b
Mongolia_1i
Mongolia_1c
Mongolia_1e
Mongolia_1d
Mongolia_1b
France_5c
Ukraine_2f
Mongolia_2a
Russia_Yakutia_2e
France_3d
Mongolia_1a
France_3c
USA_Alaska_1a
Russia_Yakutia_2a
Russia_Yakutia_2c
Russia_Yakutia_2d
Russia_Yakutia_2b
Russia_Primorski_5n
Russia_Khabarovsk_4a
Russia_Khabarovsk_3b
Russia_Primorski_5l
Russia_Primorski_5m
Russia_Sakhalin_1b
Russia_Khabarovsk_7b
Russia_Khabarovsk_7a
Czechia_1a_Daphnia_hrbaceki
Japan_1c
Japan_1b
Japan_1a
Russia_Primorski_6c
Russia_Primorski_6d
Russia_Primorski_5g
Russia_Primorski_5h
Russia_Primorski_6a
Russia_Khabarovsk_3a
Russia_Primorski_2b
Russia_Primorski_6b
Russia_Khabarovsk_3c
Russia_Primorski_2a
Russia_Khabarovsk_1a
Russia_Khabarovsk_2a
Russia_Khabarovsk_2c
Russia_Khabarovsk_2e
Russia_Khabarovsk_8a
Russia_Khabarovsk_2d
Russia_Khabarovsk_2b
Russia_Khabarovsk_2f
Russia_Primorski_5e
Russia_Primorski_5f
Russia_Primorski_1a
Russia_Primorski_4a
Russia_Primorski_5b
Russia_Primorski_5a
Russia_Primorski_5d
Russia_Primorski_5i
Russia_Primorski_5k
Russia_Primorski_5c
Russia_Primorski_5j
Russia_Sakhalin_7b
Russia_Sakhalin_7a
Russia_Sakhalin_3c
Russia_Sakhalin_5a
Russia_Sakhalin_3b
Russia_Sakhalin_3a
Russia_Sakhalin_2g
Russia_Sakhalin_2f
Russia_Sakhalin_2e
Russia_Sakhalin_2h
Russia_Sakhalin_6a
Russia_Sakhalin_2d
Russia_Sakhalin_2c
Russia_Sakhalin_2a
Russia_Sakhalin_2b
Russia_Sakhalin_1a
Russia_Sakhalin_3d
Russia_Khabarovsk_1b
Russia_Primorski_4b
Russia_Primorski_3a
SouthKorea_1h
SouthKorea_1c
SouthKorea_1g
SouthKorea_1d
SouthKorea_1i
SouthKorea_1e
SouthKorea_1f
SouthKorea_1a
SouthKorea_1b
Russia_Khabarovsk_5c
Russia_Khabarovsk_5a
Russia_Khabarovsk_5d
Russia_Khabarovsk_5b
Russia_Khabarovsk_6a
Japan_2b
Japan_2a
SouthKorea_Jeju_3a
SouthKorea_Jeju_4f
SouthKorea_Jeju_1a
SouthKorea_Jeju_4c
SouthKorea_Jeju_1b
SouthKorea_Jeju_1c
SouthKorea_Jeju_2a
SouthKorea_Jeju_2b
SouthKorea_Jeju_4b
SouthKorea_Jeju_4a
SouthKorea_Jeju_4e
SouthKorea_Jeju_4d
1
1
0.99
0.93
0.90
0.99
0.94
1
0.99
0.74
0.93
0.99
0.99
1
0.94
0.91
0.84
0.1

Supplementary Figure S3. Maximum Likelihood phylogram of the ND2 region from the *Daphnia curvirostris* complex based on an amino acid alignment with more closely related outgroup sequences added (*D.galeata* and *D. umbra*). Support values are given on the branches (Approximate LRT's). Colors represent major clades as given in Figure1.

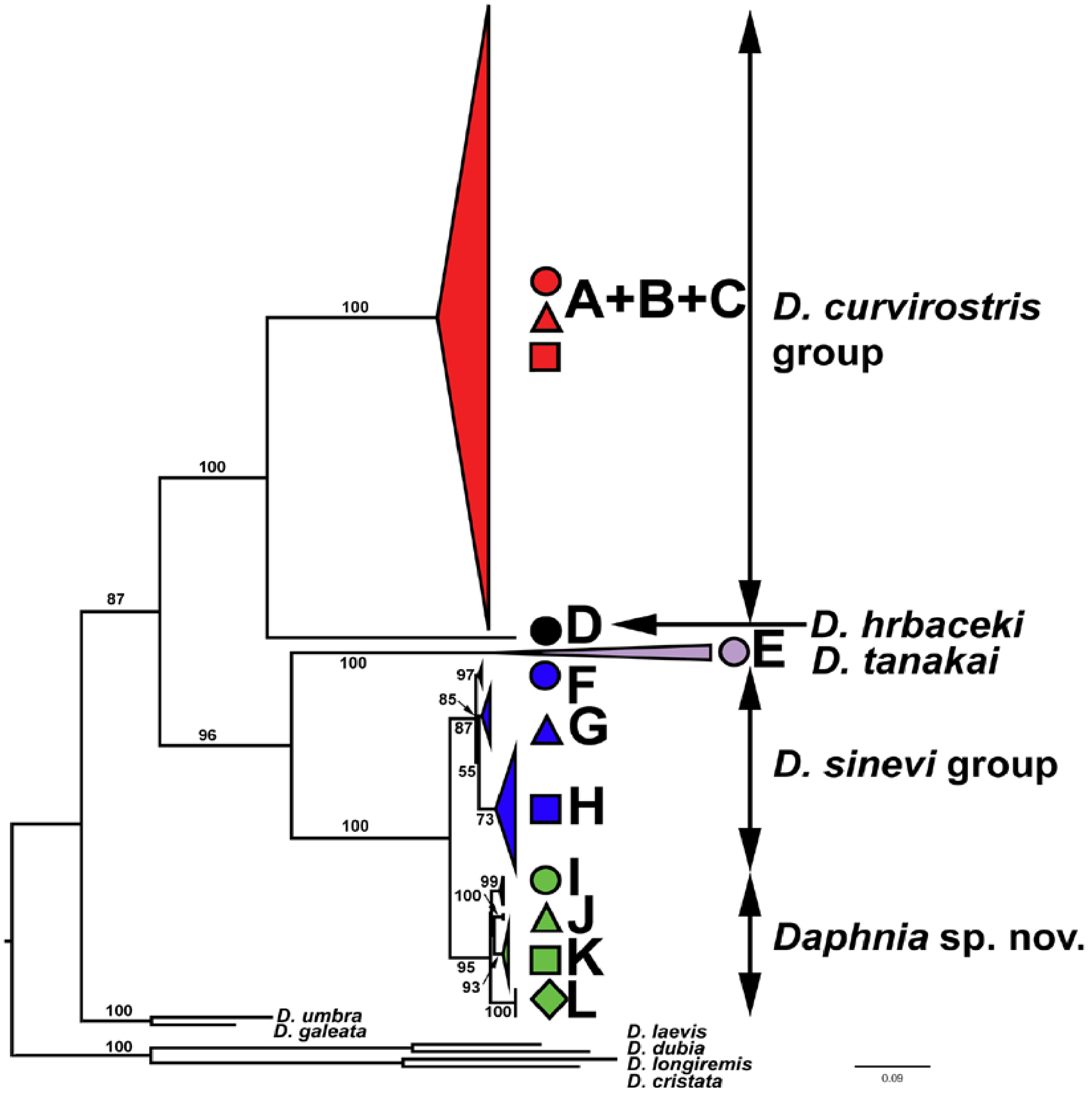

Supplementary Figure S4. The mismatch distribution analysis for *Daphnia curvirostris* based on ND2 sequences where green expected values are from a constant population size model.

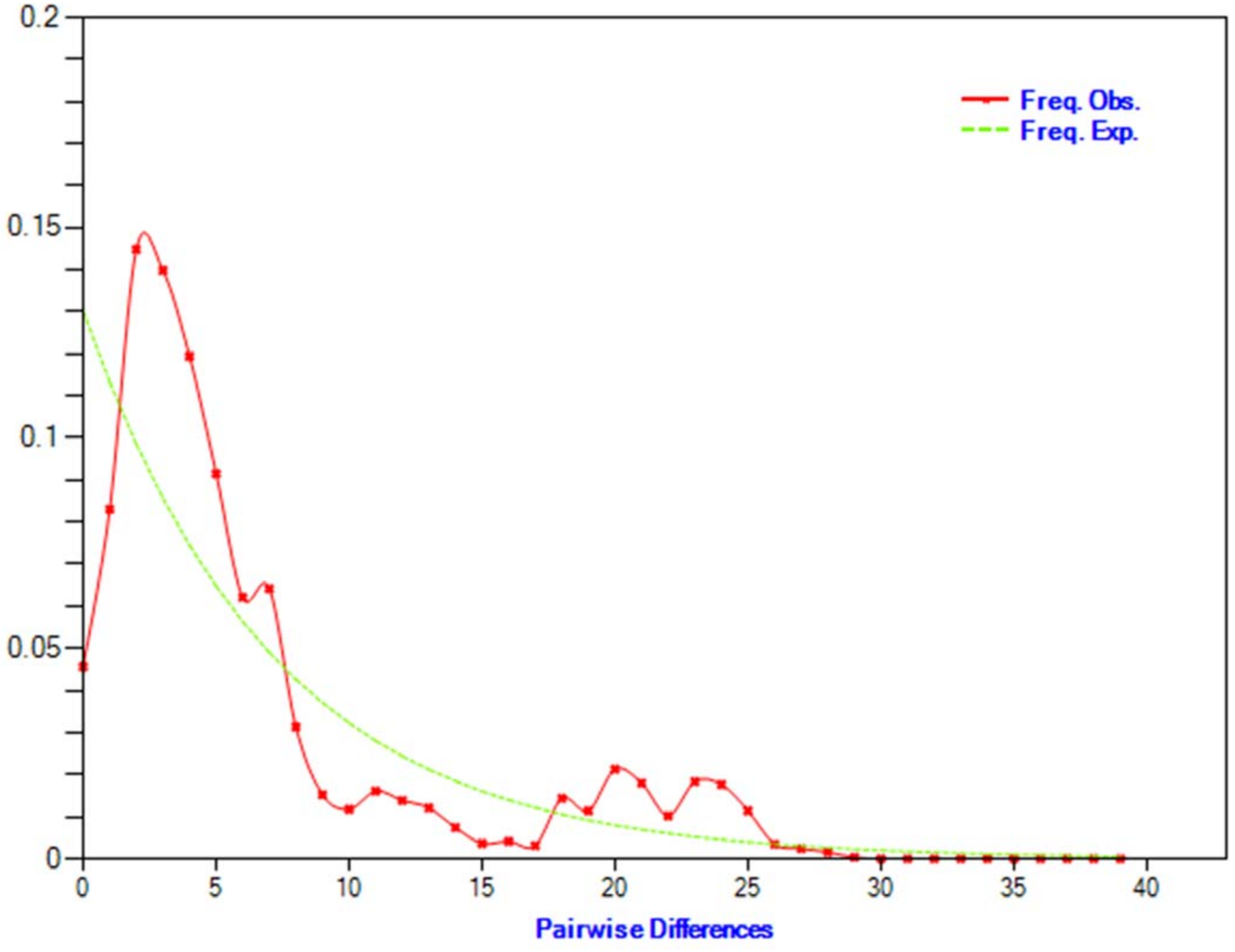

Supplementary Figure S5. Results of the Mixed Effects Model of Evolution (MEME) test for selection in the ND2 gene of the *Daphnia curvirostris* species group. Bars represent significance for episodic positive selection by codon site.

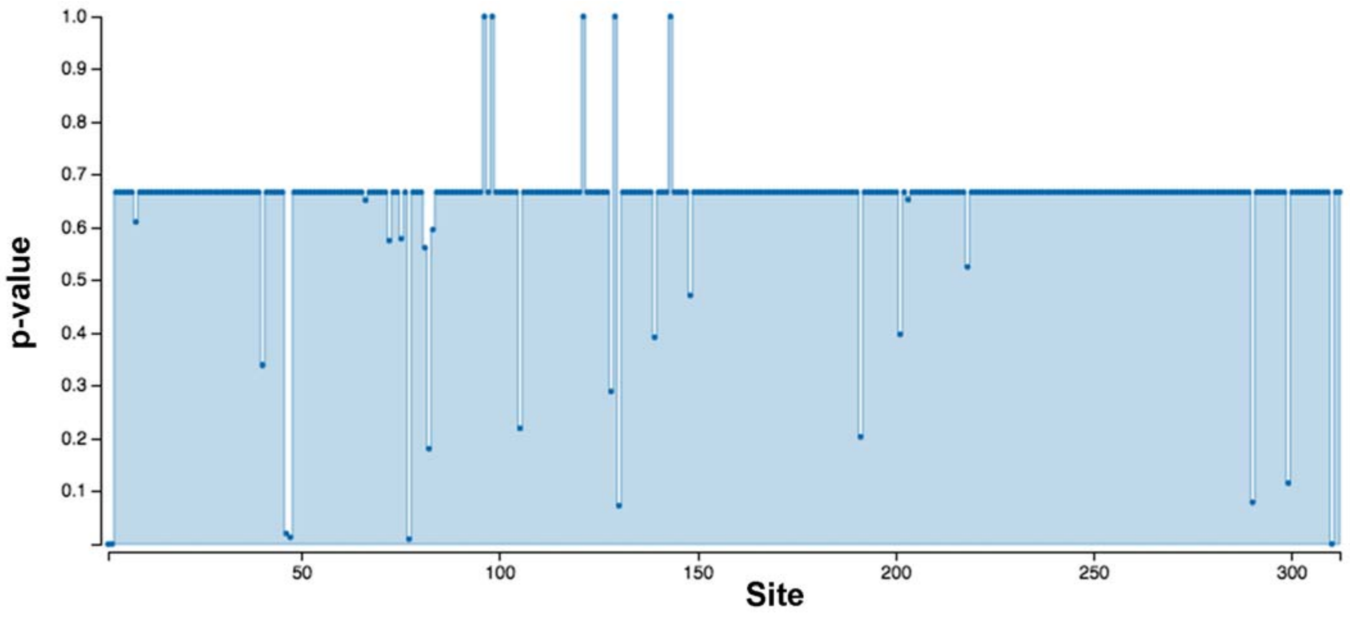

Supplementary Table 2. Results of Fast Unconstrained Bayesian AppRoximation (FUBAR) for selection at the ND2 gene of the *Daphnia curvirostris* complex. Nearly all of the significant values (174 codons) are purifying (negative) selection.

Site	Partition	&alpha	&beta	β-&alpha	Prob[α>&beta]	Prob[α<&beta]	BayesFactor [α<&beta]	PSRF	Neff
1	1	6.739	43.684	36.945	0.000	0.993	497.873	1.011	162.757
2	1	2.254	19.885	17.632	0.002	0.973	119.409	0.997	1194.870
3	1	1.103	0.351	-0.753	0.809	0.146	0.567	0.996	4445.326
4	1	2.111	0.523	-1.588	0.934	0.046	0.159	0.996	1673.760
5	1	2.781	0.571	-2.211	0.993	0.004	0.012	0.999	662.228
6	1	1.300	0.053	-1.247	0.990	0.005	0.018	0.999	562.286
7	1	1.771	0.473	-1.298	0.884	0.087	0.318	0.997	1338.889
8	1	1.770	1.362	-0.408	0.503	0.386	2.087	1.029	77.081
9	1	0.891	0.349	-0.542	0.848	0.110	0.410	0.996	2010.780
10	1	1.905	0.040	-1.865	0.998	0.001	0.004	1.004	280.880
11	1	2.714	0.264	-2.450	0.999	0.001	0.002	1.054	45.724
12	1	1.119	0.698	-0.421	0.607	0.317	1.541	0.999	637.397
13	1	1.902	0.369	-1.534	0.971	0.018	0.061	1.011	156.345
14	1	1.889	0.368	-1.521	0.970	0.019	0.064	1.003	329.116
15	1	1.345	0.680	-0.665	0.734	0.197	0.813	0.999	572.219
16	1	2.909	0.029	-2.880	1.000	0.000	0.000	0.998	842.424
17	1	0.899	0.069	-0.830	0.983	0.010	0.033	0.999	603.602
18	1	3.565	0.242	-3.323	1.000	0.000	0.000	1.048	50.626
19	1	1.301	0.059	-1.242	0.994	0.003	0.011	1.001	388.816
20	1	2.722	0.048	-2.675	1.000	0.000	0.000	0.998	757.630
21	1	2.606	0.038	-2.568	0.999	0.000	0.001	1.007	215.124
22	1	3.042	0.650	-2.392	0.991	0.005	0.016	0.998	877.031
23	1	2.250	0.332	-1.917	0.988	0.007	0.024	1.010	174.746
24	1	2.573	0.051	-2.522	0.999	0.000	0.001	1.001	398.065
25	1	2.157	0.648	-1.509	0.897	0.073	0.262	0.996	1777.567
26	1	2.568	0.024	-2.543	1.000	0.000	0.000	1.006	221.273
27	1	3.118	0.078	-3.040	1.000	0.000	0.000	1.003	323.913
28	1	2.007	0.030	-1.977	0.998	0.001	0.003	1.002	338.910
29	1	2.674	0.069	-2.605	0.999	0.000	0.001	0.999	711.545
30	1	2.519	0.023	-2.496	1.000	0.000	0.000	1.011	160.120
31	1	2.648	0.024	-2.624	1.000	0.000	0.000	1.008	202.159
32	1	2.359	0.085	-2.274	0.997	0.002	0.005	0.999	578.088
33	1	1.418	0.195	-1.223	0.981	0.011	0.037	1.005	258.966
34	1	2.215	0.047	-2.168	0.997	0.002	0.007	1.006	230.996
35	1	4.036	0.758	-3.278	0.991	0.004	0.012	1.007	216.756
36	1	2.290	0.032	-2.258	1.000	0.000	0.001	1.007	218.017
37	1	2.361	0.654	-1.707	0.931	0.047	0.163	0.998	913.780
38	1	2.724	0.355	-2.369	0.998	0.001	0.004	1.026	85.184
39	1	2.519	0.058	-2.461	0.998	0.001	0.005	1.001	432.654
40	1	1.092	0.966	-0.127	0.372	0.549	4.044	1.008	198.471
41	1	1.108	0.312	-0.796	0.843	0.116	0.435	0.995	5280.373
42	1	1.673	0.042	-1.631	0.995	0.003	0.009	1.001	428.457

43	1	2.398	0.315	-2.083	0.992	0.005	0.016	1.013	140.561
44	1	1.379	0.049	-1.329	0.995	0.002	0.008	1.002	377.637
45	1	9.002	0.427	-8.575	1.000	0.000	0.000	1.006	226.205
46	1	1.556	0.630	-0.927	0.839	0.110	0.412	0.997	1053.273
47	1	0.266	0.755	0.489	0.079	0.893	27.653	0.997	1089.987
48	1	2.451	0.282	-2.169	0.989	0.006	0.022	1.013	143.975
49	1	1.524	0.247	-1.277	0.974	0.017	0.056	1.007	215.103
50	1	1.268	0.064	-1.204	0.987	0.008	0.025	1.000	538.781
51	1	2.575	0.252	-2.323	0.995	0.003	0.010	1.052	47.084
52	1	3.015	0.025	-2.989	1.000	0.000	0.000	1.002	361.761
53	1	3.290	0.038	-3.252	1.000	0.000	0.000	0.998	718.646
54	1	2.547	0.039	-2.508	0.999	0.000	0.001	1.006	225.304
55	1	2.513	0.083	-2.430	0.993	0.006	0.019	0.999	555.440
56	1	1.345	0.207	-1.139	0.980	0.012	0.040	1.002	357.903
57	1	2.768	0.040	-2.728	1.000	0.000	0.000	1.003	297.985
58	1	2.729	0.022	-2.707	1.000	0.000	0.000	1.004	266.496
59	1	2.692	0.067	-2.625	0.999	0.000	0.001	0.998	772.369
60	1	1.762	0.035	-1.727	0.998	0.001	0.003	1.003	321.720
61	1	2.908	0.029	-2.879	1.000	0.000	0.000	0.998	847.145
62	1	2.601	0.397	-2.204	0.992	0.005	0.015	1.006	226.333
63	1	1.804	0.863	-0.941	0.691	0.241	1.056	1.015	130.725
64	1	1.813	0.388	-1.425	0.931	0.049	0.172	0.999	596.150
65	1	2.264	0.382	-1.882	0.963	0.026	0.089	1.001	389.527
66	1	2.592	1.943	-0.649	0.508	0.124	0.472	1.014	132.585
67	1	2.814	0.021	-2.793	1.000	0.000	0.000	0.998	814.897
68	1	2.734	0.022	-2.712	1.000	0.000	0.000	1.002	351.578
69	1	1.065	0.116	-0.949	0.969	0.021	0.071	1.000	465.329
70	1	2.631	1.036	-1.595	0.893	0.057	0.201	1.025	86.317
71	1	4.697	0.948	-3.749	0.992	0.001	0.003	1.009	180.389
72	1	0.750	0.694	-0.056	0.451	0.469	2.936	0.998	816.713
73	1	2.276	0.837	-1.439	0.881	0.077	0.279	1.016	121.917
74	1	2.814	0.897	-1.917	0.905	0.061	0.217	1.018	112.476
75	1	0.781	0.805	0.024	0.364	0.545	3.976	0.999	581.638
76	1	2.696	1.095	-1.601	0.886	0.056	0.195	1.029	76.201
77	1	0.822	1.206	0.384	0.196	0.717	8.410	1.003	315.327
78	1	3.139	2.058	-1.081	0.515	0.083	0.300	1.011	162.184
79	1	2.725	1.051	-1.674	0.889	0.057	0.202	1.020	104.831
80	1	3.011	0.674	-2.337	0.997	0.001	0.005	1.000	525.914
81	1	2.045	2.420	0.375	0.233	0.436	2.564	1.010	167.462
82	1	0.647	0.910	0.263	0.232	0.710	8.114	0.998	916.524
83	1	1.231	1.195	-0.036	0.373	0.521	3.606	1.009	177.616
84	1	2.253	0.035	-2.218	0.998	0.001	0.003	1.007	215.410
85	1	0.931	0.566	-0.365	0.614	0.320	1.562	0.997	1533.220
86	1	2.220	1.376	-0.845	0.670	0.186	0.760	1.018	111.057
87	1	2.726	0.022	-2.704	1.000	0.000	0.000	1.006	227.671
88	1	2.624	0.483	-2.142	0.977	0.014	0.048	1.006	237.621
89	1	2.293	0.740	-1.552	0.903	0.066	0.234	1.007	219.647
90	1	3.334	0.238	-3.096	0.999	0.001	0.002	1.052	47.045
91	1	2.221	0.033	-2.188	0.998	0.001	0.004	1.005	251.702
92	1	2.757	0.071	-2.686	1.000	0.000	0.000	0.999	579.223
93	1	1.629	0.202	-1.427	0.992	0.005	0.015	1.016	121.996
94	1	7.122	0.320	-6.802	1.000	0.000	0.000	1.004	285.571
95	1	2.717	0.020	-2.697	1.000	0.000	0.000	1.007	217.124
96	1	0.747	0.065	-0.682	0.962	0.021	0.070	0.996	2933.931
97	1	2.171	0.048	-2.123	0.996	0.002	0.007	1.005	243.000
98	1	1.577	0.041	-1.535	0.990	0.005	0.018	0.998	823.550

99	1	0.867	0.228	-0.639	0.922	0.051	0.180	0.996	1674.408
100	1	2.745	0.047	-2.698	1.000	0.000	0.000	0.999	629.488
101	1	2.886	0.766	-2.120	0.992	0.002	0.007	1.006	232.143
102	1	6.977	0.815	-6.162	1.000	0.000	0.000	1.002	344.021
103	1	0.559	0.488	-0.070	0.501	0.422	2.426	0.998	790.378
104	1	1.949	0.247	-1.702	0.987	0.008	0.027	1.027	80.318
105	1	1.061	0.192	-0.868	0.964	0.022	0.074	0.997	1528.484
106	1	3.412	0.196	-3.217	1.000	0.000	0.000	1.065	39.434
107	1	2.477	0.498	-1.979	0.982	0.011	0.036	0.998	853.424
108	1	2.800	0.068	-2.732	1.000	0.000	0.000	1.001	392.936
109	1	3.227	0.338	-2.888	1.000	0.000	0.000	1.014	135.280
110	1	2.755	0.049	-2.706	1.000	0.000	0.001	1.001	412.952
111	1	2.274	0.634	-1.640	0.945	0.034	0.117	0.997	1482.085
112	1	2.547	0.604	-1.943	0.974	0.016	0.053	0.995	5217.805
113	1	2.198	0.082	-2.116	0.996	0.002	0.008	1.000	533.393
114	1	2.229	0.047	-2.182	0.997	0.002	0.006	1.006	231.190
115	1	2.856	0.867	-1.989	0.960	0.023	0.077	1.019	109.217
116	1	2.274	0.083	-2.192	0.996	0.002	0.008	0.999	576.159
117	1	3.381	0.077	-3.305	1.000	0.000	0.000	1.001	404.589
118	1	2.158	0.031	-2.126	0.999	0.000	0.001	1.006	224.209
119	1	3.061	0.678	-2.383	0.973	0.018	0.060	1.000	487.723
120	1	2.101	0.052	-2.048	0.995	0.003	0.011	1.005	264.084
121	1	0.660	0.062	-0.598	0.824	0.091	0.332	1.008	188.164
122	1	2.067	0.242	-1.824	0.988	0.008	0.026	1.032	71.295
123	1	3.021	0.022	-2.999	1.000	0.000	0.000	1.008	191.449
124	1	2.250	0.033	-2.216	0.999	0.000	0.001	1.006	221.666
125	1	2.791	0.051	-2.740	1.000	0.000	0.000	0.999	616.941
126	1	2.567	0.374	-2.193	0.981	0.013	0.043	1.024	90.718
127	1	2.633	0.621	-2.012	0.981	0.010	0.035	0.997	1186.651
128	1	1.126	0.991	-0.135	0.395	0.497	3.284	1.007	216.391
129	1	1.956	0.067	-1.889	0.992	0.005	0.017	1.003	326.483
130	1	1.908	0.747	-1.161	0.804	0.145	0.561	1.006	229.568
131	1	2.963	0.037	-2.926	1.000	0.000	0.000	1.000	493.699
132	1	1.872	0.764	-1.107	0.815	0.130	0.494	1.008	202.275
133	1	3.673	0.876	-2.797	0.996	0.001	0.003	1.014	138.069
134	1	2.836	0.904	-1.932	0.982	0.008	0.027	1.021	101.180
135	1	2.472	0.778	-1.693	0.918	0.053	0.187	1.013	143.961
136	1	3.780	0.565	-3.214	1.000	0.000	0.000	1.000	494.355
137	1	2.300	1.173	-1.127	0.741	0.165	0.656	1.032	70.217
138	1	1.846	0.847	-0.998	0.748	0.186	0.760	1.012	153.114
139	1	0.827	0.423	-0.405	0.713	0.228	0.982	0.996	1980.962
140	1	2.494	0.926	-1.568	0.881	0.078	0.281	1.022	96.233
141	1	2.196	0.786	-1.410	0.831	0.125	0.476	1.010	168.441
142	1	2.973	0.607	-2.366	0.986	0.008	0.027	0.996	1881.623
143	1	1.273	0.635	-0.638	0.560	0.397	2.183	1.000	530.519
144	1	1.807	0.592	-1.214	0.843	0.118	0.445	0.996	3335.477
145	1	2.605	0.732	-1.874	0.965	0.020	0.069	1.004	267.805
146	1	3.587	0.255	-3.331	1.000	0.000	0.000	1.041	58.389
147	1	2.956	0.067	-2.889	1.000	0.000	0.000	1.000	462.000
148	1	2.091	1.317	-0.774	0.636	0.251	1.110	1.035	66.410
149	1	2.717	0.560	-2.157	0.992	0.004	0.013	0.996	2281.464
150	1	5.462	0.047	-5.416	1.000	0.000	0.000	0.998	802.931
151	1	2.681	0.203	-2.479	1.000	0.000	0.000	1.071	36.523
152	1	1.709	0.589	-1.120	0.894	0.066	0.236	1.000	501.759
153	1	2.790	0.778	-2.012	0.990	0.004	0.014	1.008	190.959
154	1	1.814	0.037	-1.777	0.999	0.000	0.001	1.004	273.973

155	1	7.864	0.783	-7.081	0.999	0.000	0.000	1.004	290.100
156	1	2.830	0.903	-1.927	0.981	0.009	0.029	1.021	97.904
157	1	3.059	0.041	-3.018	1.000	0.000	0.000	0.998	875.077
158	1	2.356	0.028	-2.328	1.000	0.000	0.001	1.008	192.374
159	1	2.601	0.028	-2.574	1.000	0.000	0.000	1.010	174.015
160	1	2.731	0.464	-2.267	0.991	0.005	0.017	1.000	539.192
161	1	2.015	0.042	-1.972	0.998	0.001	0.004	1.005	262.405
162	1	2.246	0.027	-2.219	1.000	0.000	0.000	1.008	201.841
163	1	1.588	0.117	-1.471	0.984	0.010	0.035	1.001	437.098
164	1	1.635	0.715	-0.920	0.771	0.169	0.675	1.002	371.236
165	1	1.144	0.206	-0.938	0.973	0.016	0.054	0.997	1014.897
166	1	2.089	0.049	-2.040	0.996	0.002	0.008	1.005	259.733
167	1	1.809	0.047	-1.762	0.998	0.001	0.005	1.003	316.832
168	1	0.871	0.075	-0.796	0.976	0.014	0.047	0.999	657.198
169	1	2.655	0.029	-2.627	1.000	0.000	0.000	1.011	162.522
170	1	2.812	0.021	-2.791	1.000	0.000	0.000	1.012	148.995
171	1	2.642	0.024	-2.618	1.000	0.000	0.000	1.012	151.898
172	1	2.434	0.034	-2.400	1.000	0.000	0.001	1.004	292.200
173	1	1.281	0.047	-1.235	0.998	0.001	0.004	1.003	319.158
174	1	3.006	0.051	-2.955	1.000	0.000	0.000	1.001	398.401
175	1	2.932	0.052	-2.880	1.000	0.000	0.000	1.000	489.303
176	1	2.598	0.074	-2.524	0.999	0.001	0.002	0.999	686.906
177	1	3.301	0.603	-2.698	0.999	0.001	0.002	0.997	1387.882
178	1	2.842	0.025	-2.817	1.000	0.000	0.000	1.000	533.831
179	1	2.744	0.798	-1.945	0.975	0.011	0.038	1.008	192.839
180	1	2.040	0.780	-1.260	0.815	0.136	0.522	1.010	174.308
181	1	2.059	0.692	-1.368	0.873	0.089	0.326	1.000	481.924
182	1	2.223	0.570	-1.652	0.951	0.031	0.107	0.996	2849.995
183	1	1.974	0.575	-1.399	0.890	0.079	0.284	0.996	2596.170
184	1	5.967	0.650	-5.317	0.999	0.000	0.001	1.004	292.643
185	1	2.575	0.921	-1.654	0.906	0.057	0.200	1.017	115.628
186	1	2.208	0.476	-1.731	0.957	0.029	0.100	0.999	669.029
187	1	1.123	0.712	-0.410	0.621	0.304	1.447	0.999	603.242
188	1	1.514	0.840	-0.675	0.638	0.282	1.305	1.010	171.892
189	1	2.573	0.037	-2.536	0.999	0.000	0.001	1.008	197.923
190	1	2.188	0.047	-2.141	0.997	0.002	0.007	1.006	236.424
191	1	1.125	0.895	-0.230	0.439	0.474	2.996	1.007	211.053
192	1	2.533	0.486	-2.047	0.980	0.012	0.041	0.999	710.175
193	1	3.741	0.043	-3.698	1.000	0.000	0.000	0.999	677.129
194	1	1.967	0.876	-1.091	0.769	0.168	0.669	1.016	122.639
195	1	2.428	0.808	-1.620	0.908	0.060	0.212	1.014	134.544
196	1	2.679	0.759	-1.920	0.944	0.036	0.125	1.008	197.619
197	1	2.109	0.097	-2.011	0.989	0.008	0.025	0.999	596.206
198	1	2.828	0.394	-2.434	0.999	0.001	0.002	1.014	134.547
199	1	2.629	0.341	-2.288	0.997	0.002	0.006	1.014	132.548
200	1	2.735	0.025	-2.710	1.000	0.000	0.000	1.006	236.849
201	1	1.830	0.624	-1.205	0.815	0.145	0.562	0.996	2265.667
202	1	3.794	0.374	-3.420	1.000	0.000	0.000	1.012	151.440
203	1	0.838	0.512	-0.325	0.633	0.302	1.436	0.997	1361.250
204	1	2.310	0.504	-1.805	0.945	0.038	0.131	0.998	948.618
205	1	3.615	0.620	-2.994	0.987	0.008	0.027	0.997	1446.100
206	1	1.858	1.141	-0.717	0.590	0.316	1.534	1.025	85.483
207	1	2.136	0.992	-1.144	0.774	0.154	0.604	1.021	101.244
208	1	2.088	0.030	-2.058	0.999	0.001	0.002	1.004	281.591
209	1	2.840	0.623	-2.217	0.986	0.008	0.028	0.996	2522.017
210	1	2.789	0.028	-2.760	1.000	0.000	0.000	0.999	559.182

211	1	2.715	0.024	-2.691	1.000	0.000	0.000	1.012	146.027
212	1	2.737	0.680	-2.057	0.972	0.017	0.056	1.000	544.802
213	1	2.189	0.028	-2.162	0.999	0.001	0.002	1.005	253.717
214	1	2.213	0.353	-1.859	0.985	0.009	0.031	1.008	200.897
215	1	2.064	0.065	-2.000	0.998	0.001	0.005	1.001	425.600
216	1	2.721	0.025	-2.696	1.000	0.000	0.000	1.003	311.915
217	1	2.866	0.637	-2.230	0.988	0.007	0.023	0.998	873.121
218	1	1.016	1.084	0.067	0.301	0.612	5.245	1.007	209.984
219	1	2.535	1.770	-0.765	0.567	0.178	0.717	1.024	89.230
220	1	2.698	0.665	-2.033	0.934	0.046	0.160	0.999	668.551
221	1	2.576	0.573	-2.002	0.981	0.011	0.038	0.996	3233.249
222	1	7.061	0.861	-6.200	0.999	0.000	0.000	1.000	459.252
223	1	3.244	1.593	-1.651	0.703	0.119	0.449	1.028	78.364
224	1	2.599	0.856	-1.743	0.923	0.049	0.170	1.019	108.199
225	1	0.696	0.425	-0.271	0.650	0.284	1.318	0.997	1320.566
226	1	2.321	0.924	-1.396	0.857	0.091	0.334	1.019	107.329
227	1	2.572	0.381	-2.191	0.986	0.009	0.031	1.024	88.533
228	1	3.241	0.392	-2.849	0.999	0.000	0.001	1.015	126.104
229	1	2.360	0.422	-1.937	0.974	0.017	0.057	1.001	409.698
230	1	2.927	0.037	-2.889	1.000	0.000	0.000	1.004	293.379
231	1	7.424	0.278	-7.146	1.000	0.000	0.000	1.004	267.397
232	1	1.643	0.374	-1.269	0.939	0.042	0.145	1.005	257.038
233	1	2.651	0.045	-2.606	0.999	0.000	0.001	1.002	370.721
234	1	2.732	0.031	-2.701	1.000	0.000	0.000	0.999	627.398
235	1	2.664	0.072	-2.592	1.000	0.000	0.001	0.999	612.311
236	1	1.137	0.050	-1.088	0.997	0.002	0.005	1.003	318.557
237	1	2.670	0.023	-2.648	1.000	0.000	0.000	1.004	276.916
238	1	3.910	0.076	-3.833	1.000	0.000	0.000	1.001	395.456
239	1	2.717	0.025	-2.692	1.000	0.000	0.000	1.003	302.267
240	1	2.801	0.024	-2.777	1.000	0.000	0.000	1.000	491.207
241	1	1.687	0.060	-1.627	0.992	0.005	0.016	1.002	382.336
242	1	2.712	0.075	-2.637	1.000	0.000	0.000	1.000	529.199
243	1	2.729	0.022	-2.707	1.000	0.000	0.000	1.001	405.234
244	1	2.622	0.038	-2.584	1.000	0.000	0.001	1.004	284.774
245	1	3.394	0.559	-2.835	0.999	0.000	0.001	1.001	453.445
246	1	3.077	0.027	-3.049	1.000	0.000	0.000	0.998	750.762
247	1	2.925	0.024	-2.901	1.000	0.000	0.000	1.006	240.208
248	1	2.712	0.049	-2.663	1.000	0.000	0.001	1.001	405.283
249	1	2.637	0.625	-2.012	0.957	0.029	0.098	0.996	3064.427
250	1	2.536	0.510	-2.026	0.973	0.018	0.061	0.997	1063.989
251	1	2.227	0.060	-2.167	0.995	0.004	0.012	1.004	270.216
252	1	0.634	0.093	-0.541	0.786	0.131	0.499	1.008	194.087
253	1	1.451	0.585	-0.866	0.776	0.173	0.696	0.996	2408.613
254	1	1.982	0.961	-1.021	0.720	0.210	0.880	1.018	111.213
255	1	2.738	0.796	-1.942	0.944	0.036	0.123	1.012	148.600
256	1	1.485	0.940	-0.545	0.552	0.362	1.887	1.013	138.850
257	1	1.616	0.770	-0.846	0.664	0.273	1.249	1.005	241.750
258	1	1.469	0.608	-0.861	0.809	0.139	0.537	0.996	1703.284
259	1	2.313	0.562	-1.752	0.940	0.042	0.144	0.996	4468.535
260	1	3.086	0.676	-2.410	0.990	0.006	0.019	1.000	497.109
261	1	2.086	0.864	-1.222	0.775	0.170	0.679	1.018	112.794
262	1	3.393	0.542	-2.850	0.999	0.000	0.001	1.000	466.574
263	1	2.355	0.249	-2.106	0.995	0.003	0.009	1.044	54.731
264	1	2.614	0.551	-2.063	0.983	0.010	0.032	0.998	752.700
265	1	2.630	1.328	-1.302	0.793	0.075	0.269	1.032	71.552
266	1	2.844	0.235	-2.609	0.994	0.003	0.011	1.042	56.333

267	1	2.949	0.254	-2.695	0.997	0.002	0.006	1.054	46.088
268	1	1.856	0.809	-1.047	0.787	0.149	0.580	1.008	201.709
269	1	2.005	0.358	-1.647	0.970	0.020	0.067	1.003	324.848
270	1	2.888	0.025	-2.863	1.000	0.000	0.000	0.998	746.019
271	1	2.519	0.077	-2.442	0.998	0.001	0.003	0.999	693.938
272	1	1.678	0.551	-1.128	0.842	0.119	0.448	0.996	3327.530
273	1	1.950	0.035	-1.914	0.999	0.001	0.002	1.004	270.999
274	1	2.660	0.051	-2.610	1.000	0.000	0.001	0.999	707.913
275	1	1.000	0.283	-0.717	0.862	0.101	0.372	0.996	3533.577
276	1	1.699	0.268	-1.432	0.961	0.026	0.089	1.010	171.694
277	1	2.535	0.037	-2.499	0.999	0.000	0.001	1.007	205.156
278	1	2.798	0.049	-2.749	1.000	0.000	0.000	0.999	644.612
279	1	1.990	0.042	-1.948	0.999	0.000	0.002	1.004	294.740
280	1	2.327	0.354	-1.973	0.986	0.008	0.027	1.022	95.071
281	1	2.224	0.036	-2.188	0.998	0.001	0.004	1.006	230.131
282	1	2.560	0.039	-2.520	0.999	0.000	0.001	1.007	209.430
283	1	2.575	0.695	-1.880	0.969	0.018	0.061	1.001	446.129
284	1	2.266	0.416	-1.850	0.980	0.012	0.041	1.001	386.153
285	1	0.695	0.086	-0.608	0.826	0.104	0.385	1.007	204.454
286	1	2.644	0.029	-2.615	1.000	0.000	0.000	1.011	162.708
287	1	1.661	0.267	-1.394	0.959	0.028	0.095	1.008	198.047
288	1	1.050	0.052	-0.998	0.994	0.003	0.011	1.001	393.830
289	1	2.505	0.463	-2.043	0.989	0.006	0.020	1.000	537.482
290	1	1.798	1.251	-0.548	0.559	0.332	1.647	1.023	91.868
291	1	1.759	0.491	-1.268	0.896	0.075	0.271	0.998	978.064
292	1	2.289	0.619	-1.670	0.925	0.052	0.180	0.995	5088.296
293	1	2.358	0.816	-1.541	0.895	0.069	0.245	1.013	144.820
294	1	3.414	1.184	-2.230	0.911	0.023	0.077	1.024	90.860
295	1	1.947	0.799	-1.148	0.775	0.171	0.686	1.013	144.858
296	1	2.726	1.446	-1.280	0.745	0.090	0.328	1.024	90.612
297	1	4.742	0.887	-3.855	0.993	0.003	0.009	1.013	145.003
298	1	3.396	1.353	-2.042	0.861	0.006	0.020	1.012	152.222
299	1	2.235	2.036	-0.199	0.412	0.312	1.504	1.023	93.456
300	1	8.228	0.984	-7.244	0.999	0.000	0.000	1.001	394.112
301	1	2.539	0.476	-2.063	0.986	0.008	0.027	0.999	587.985
302	1	5.678	0.945	-4.733	0.994	0.000	0.001	1.003	296.837
303	1	2.808	0.705	-2.102	0.947	0.036	0.122	1.002	361.467
304	1	4.526	0.883	-3.643	0.993	0.001	0.004	1.009	184.119
305	1	2.665	0.869	-1.796	0.924	0.048	0.169	1.019	107.427
306	1	3.974	1.003	-2.970	0.970	0.008	0.027	1.016	121.849
307	1	2.882	0.523	-2.359	0.996	0.002	0.008	0.999	643.554
308	1	1.052	0.468	-0.585	0.781	0.168	0.671	0.996	2554.527
309	1	3.425	0.741	-2.684	0.997	0.001	0.004	1.006	233.861
310	1	0.711	3.579	2.868	0.004	0.957	73.154	1.001	417.710
311	1	5.114	0.899	-4.215	0.996	0.001	0.003	1.008	192.367
312	1	4.653	12.338	7.684	0.337	0.600	4.981	1.012	146.717
